# Whole Exome Sequencing of HIV-1 long-term non-progressors identifies rare variants in genes encoding innate immune sensors and signaling molecules

**DOI:** 10.1038/s41598-018-33481-0

**Published:** 2018-10-15

**Authors:** Sara Konstantin Nissen, Mette Christiansen, Marie Helleberg, Kathrine Kjær, Sofie Eg Jørgensen, Jan Gerstoft, Terese L Katzenstein, Thomas Benfield, Gitte Kronborg, Carsten S Larsen, Alex Laursen, Gitte Pedersen, Martin R Jakobsen, Martin Tolstrup, Trine H Mogensen

**Affiliations:** 1 Department of Infectious Diseases, Aarhus University Hospital, Palle Juul-Jensens Boulevard 99, 8200 Aarhus N, Denmark; 2 Department of Biomedicine, Aarhus University, Wilhelm Meyers Alle 4, 8000 Aarhus C, Denmark; 3 Department of Clinical Immunology, Aarhus University Hospital, Palle Juul-Jensens Boulevard 99, 8200 Aarhus N, Denmark; 4 Department of Infectious Diseases, Copenhagen University Hospital, Rigshospitalet, Blegdamsvej 9, 2100 Copenhagen Ø, Denmark; 5 Center of Excellence for Health Immunity and Infections, Department of Infectious Diseases, Copenhagen University Hospital, Rigshospitalet, Blegdamsvej 9, 2100 Copenhagen Ø, Denmark; 6 Department of Clinical Medicine, University of Copenhagen, 2000 Copenhagen N, Denmark; 7 Department of Infectious Diseases, Hvidovre Hospital, Kettegaard Alle 30, 2650 Hvidovre, Denmark; 8 Department of Infectious Diseases, Aalborg University Hospital, Mølleparkvej 4, 9000 Aalborg, Denmark; 9 Department of Clinical Medicine, Aarhus University, Palle Juul-Jensens Boulevard, 8200 Aarhus N, Denmark

## Abstract

Common *CCR5*-∆32 and HLA alleles only explain a minority of the HIV long-term non-progressor (LTNP) and elite controller (EC) phenotypes. To identify rare genetic variants contributing to the slow disease progression phenotypes, we performed whole exome sequencing (WES) on seven LTNPs and four ECs. HLA and *CCR5* allele status, total HIV DNA reservoir size, as well as variant-related functional differences between the ECs, LTNPs, and eleven age- and gender-matched HIV-infected non-controllers on antiretroviral therapy (NCARTs) were investigated. Several rare variants were identified in genes involved in innate immune sensing, CD4-dependent infectivity, HIV trafficking, and HIV transcription mainly within the LTNP group. ECs and LTNPs had a significantly lower HIV reservoir compared to NCARTs. Furthermore, three LTNPs with variants affecting HIV nuclear import showed integrated HIV DNA levels below detection limit after *in vitro* infection. HIV slow progressors with variants in the TLR and NOD2 pathways showed reduced pro-inflammatory responses compared to matched controls. Low-range plasma levels of fibronectin was observed in a LTNP harboring two *FN1* variants. Taken together, this study identified rare variants in LTNPs as well as in one EC, which may contribute to understanding of HIV pathogenesis and these slow progressor phenotypes, especially in individuals without protecting *CCR5*-∆32 and HLA alleles.

## Introduction

HIV infection results in a diverse clinical spectrum regarding disease progression and the development of progressive cellular immunodeficiency. Some of the rare extreme phenotypes are known as elite controllers (ECs) and long-term non-progressors (LTNPs), whereas most HIV-infected individuals progress relatively fast without treatment and are termed non-controllers (NCs). A recent systematic review of almost 400 studies identified more than 200 unique definitions of ECs and LTNPs^1^. The heterogeneity in clinical presentations and definitions emphasizes the huge variation in the biological mechanism underlying the EC and LTNP phenotypes. Approximately 1% of HIV-infected individuals are ECs or LTNPs^2^. Neither of these groups experience opportunistic infections and also do not develop AIDS; and are often defined as having CD4+ T cell counts above 500 cells/μL for >10 years without antiretroviral treatment (ART). Furthermore, ECs also maintain plasma viral load (VL) below 100 copies/mL, whereas the VL of LTNPs is somewhat higher, but generally lower than for NCs^2^. On average, according to the natural history of HIV infection, NCs die within 7.4–11.5 years without ART^3^. The mechanisms underlying the HIV EC and LTNP phenotypes are only partially understood. However, it is known that low expression of the CCR5 co-receptor as well as a ∆32 deletion in this gene is associated with slow disease progression^4^. Nevertheless, the *CCR5*-∆32 variant does not protect against HIV disease, since several NCs are ∆32 heterozygous and some ∆32 homozygous individuals have also been infected^5^. Likewise, a V64I variant in *CCR2* has been shown to be associated with slow progression^6^, although this association has later been doubted^7^. Both variants have an allele frequency of approx. 10%, thus much too frequent to solely account for the rare EC and LTNP phenotypes^6^. Additionally, it is known that the adaptive immune response in HIV ECs and LTNPs possesses a more efficient cytotoxic CD8 + T cell killing capacity, and several protective HLA alleles have been identified^8–11^. Two genome-wide association studies (GWAS) estimated that the protective HLA- and chemokine alleles together with the additive effect of other common variants, together with gender and age, explains approximately 25% of the variability in VL and CD4 T cell decline between ECs and LTNPs versus NCs. This suggests that rare variants that cannot be detected in GWAS studies (due to lack of power) should be investigated in order to help understand the EC and LTNP phenotypes^10,12^.

HIV-1 disease progression has also been correlated with microbial infection and innate immune activation. Several studies have suggested a role of microbial translocation from the damaged gut mucosa in disease progression^13^. Consequently, microbial translocation activating innate pattern recognition receptors (PPRs) is believed to affect disease progression and non-AIDS comorbidities. Paradoxically, the chronic antiviral interferon (IFN) responses together with pro-inflammatory cytokine production appears to attract new target cells, enhance HIV replication, and cause exhaustion of the immune system^14^. In late-stage disease, high IFN-α plasma levels are correlated to HIV disease progression^15^. Moreover, based on studies of natural primate hosts controlling Simian immunodeficiency virus it has been hypothesized that downregulation of IFN and IFN stimulated gene (ISG) responses during the transition from acute to chronic infection may be essential for the non-progressing phenotypes of natural primate hosts and human ECs and LTNPs^14,16^. Finally, variants and epigenetic changes that increase expression of restriction factors have also been found to be elevated in HIV ECs and LTNPs^14,17^.

By investigating variants in whole exomes at the individual level, we here demonstrate that HIV slow progressors, in particular LTNPs, harbor rare variants in genes predicted to influence HIV-infectivity and replication, as well as in genes involved in mounting immune responses to HIV and microbial pathogens.

## Results

### Study population

**Figure 1:**
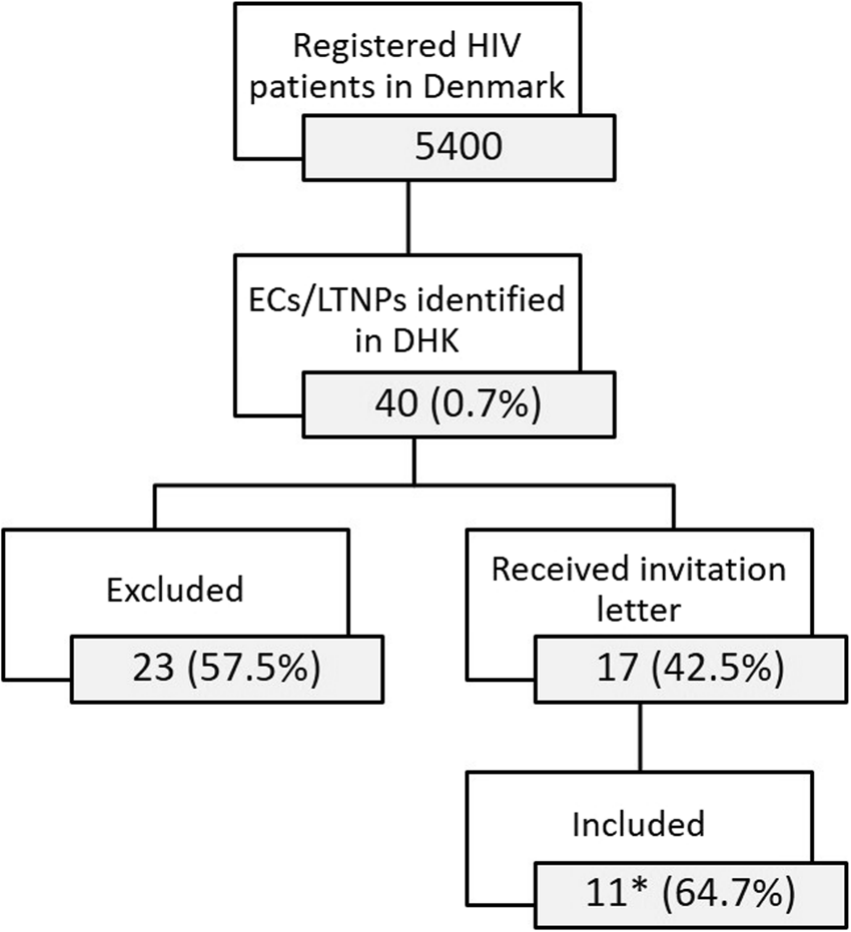
Flowchart for inclusion of ECs and LTNPs. *LTNP 011 did not fulfill all criteria due to decline in CD4 count after ten years of infection, however still controlling plasma virus for twelve more years. Elite controller (EC), long-term non-progressor (LTNP), The Danish HIV Cohort (DHK).

In 2015, 5400 HIV-infected individuals were registered in the Danish HIV Cohort (DHK). Consistent with previous studies, 0.7% of these fulfilled the criteria of belonging to the EC or LTNP groups as defined in the present study (see Methods section). More than half of the initially identified patients in the database were excluded due to recent migration, disease progression, or death; due to a two-year delay in updating of the DHK database. A total of eleven untreated HIV-infected ECs (four) and LTNPs (seven) (one LTNP did not fulfill all criteria) and a control group of eleven NCARTs were included (Fig. 1). After inclusion, the groups were age- and gender-matched (Supplementary Table 1). Table 1 shows the demographics of the EC/LTNP and NCART groups, displaying overall similar distribution between gender, ethnicity, age, and time-span between HIV diagnosis and study inclusion.

**Table 1:** Cohort characteristics.

Patients	NCART (min-max)	ECs/LTNPs (min-max)
Cohort size (N)	11	4/7**
Age*	58.18 (44–77)	57.3 (36–72)
Males (N)	7	5
Females (N)	4	6
Caucasian (N)	7	8
African (N)	4	3
Years with HIV*	18.60 (12.96–23.16)	18.38 (25.8**, 10.73–31.07)
Years on treatment*	17.73 (12.82–22.15	0 (0–0)
Nadir CD4 cells/µL	106 (40–266)	534 (160**, 343–784)
CD4 cells/µL at inclusion date	589 (80–1170)	845.7 (295**, 410–1300)
VL at inclusion date	28 (<19–50)	325.5 (<19–1987)

Numbers are shown as exact numbers (N) or mean with range. *Calculated to inclusion date. **LTNP 011 did not fulfill all criteria due to decline in CD4 count after twelve years of infection, however still controlling plasma virus for twelve more years. Non-controllers on ART (NCARTs), elite controllers (ECs), long-term non-progressors (LTNPs), viral load (VL) in RNA copies/mL.

### Reduced total HIV DNA in CD4 T cells in ECs and LTNPs

**Figure 2:**
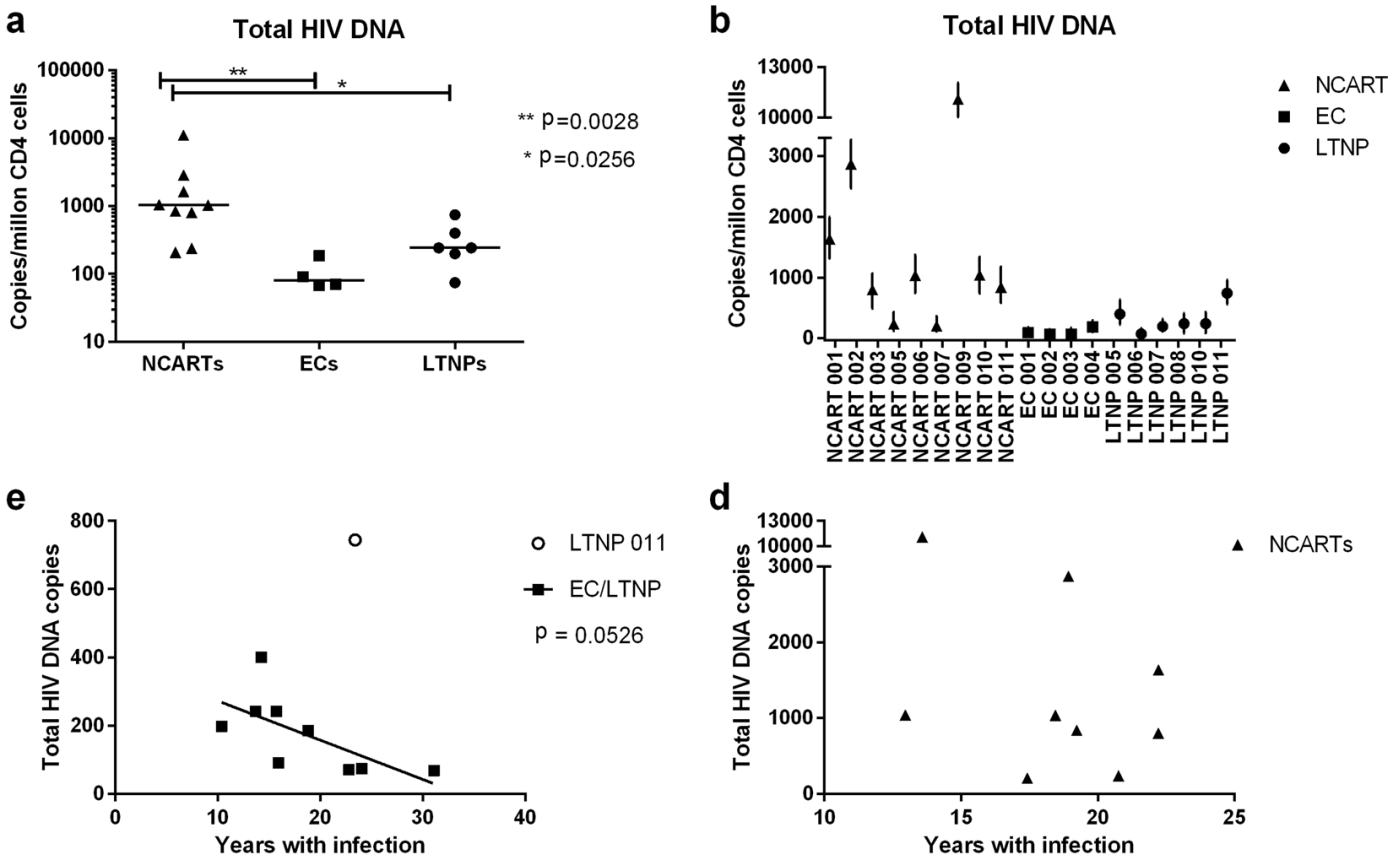
Total HIV DNA levels in CD4 T cells in ECs and LTNPs compared to NCARTs. (**a**,**b**) Total HIV DNA was measured in CD4+ T cells. NCART 004, NCART 008, and LTNP 009 were not measured for total HIV DNA due to primer-probe mismatch. (**a**) Median for each group are shown, p-values are calculated using Mann Whitney test. (**b**) DNA levels for the individual patients: Error bars represent min and max values from technical replicates. (**c**,**d**) Total HIV DNA copies per million CD4+ T cells are correlated to years with HIV infection in the EC and LTNP group (**c**) and the control group with NCARTs (**d**). The two-tailed p value from a Pearson correlation was 0.0526 for the ECs/LTNPs with R squared at 0.4368; and 0.1531 for the NCARTs with R squared at 0.2683. LTNP 011 was excluded from the analysis due to recent progression. Non-controller on ART (NCART); elite controller (EC); long-term non-progressor (LTNP).

It has previously been demonstrated that ECs display a trend towards decreased total HIV DNA in CD4+ T cells^18^. Therefore, we next wished to address this point and found significantly less total HIV DNA in CD4+ cells in both ECs (four individuals, mean 80 copies/10^6^ CD4+ cells) and LTNPs (six individuals, mean 242 copies/10^6^ CD4+ cells) compared to NCARTs (nine individuals, mean 1039 copies/10^6^ CD4+ cells) (Fig. 2a,b). We also observed a negative correlation between time since infection and total HIV DNA in CD4+ T cells from the slow progressors, although this was not significant due to the few patients included (Fig. 2c). No such trend was observed for the NCART group (Fig. 2d).

### HLA and CCR5 genotypes

Since previous studies have demonstrated an impact of HLA and *CCR5* genotypes on HIV disease progression, we wanted to examine the distribution of these in our cohort of HIV ECs and LTNPs. As shown in Table 2, five (EC 002, EC 003, LTNP 006, LTNP 009, and LTNP 011) of the eleven slow progressors were found to be heterozygous for the *CCR5*-∆32 allele, whereas two NCARTs (NCART 003 and NCART 011) also were heterozygous for this allele (Supplementary Table 1). Five of eleven slow progressors had HLA alleles associated with low risk of transmission or slow progression (EC 002, EC 003, LTNP 006, LTNP 010, and LTNP 011) (Table 2). With the exception of LTNP 006, all individuals with protective alleles also carried alleles associated with accelerated disease progression or increased transmission risk. Furthermore, high-risk HLA alleles were also present in EC 001, EC 004, EC 008, and LTNP 009 (Table 2). Altogether, five out of eleven slow progressors (EC 001, EC 004, EC 005, LTNP 007, and LTNP 008) carried neither the *CCR5*-∆32 variant nor a protective HLA subtype. Lastly, EC 004, EC 005, LTNP 006, LTNP 007, and LTNP 008 were heterozygous for *CCR2*-V64I and LTNP 010 was homozygous. However, the influence of *CCR2*-V64I on slow disease progression has been questioned for which reason this allele is not considered further in the present study^7^. No protective MHC class II alleles were identified (Supplementary Table 2).

**Table 2:** Distribution of known protective HLA and chemokine receptor alleles in ECs/LTNPs.

		MHC I subtype			Chemokine receptor genotype	
Patient	Allele	*HLA*-A	*HLA*-B	*HLA*-C	*CCR5*	*CCR2*
EC 001	1	A*11:01:01	B*07:02:01	C*04:01:01	WT	WT
	2	A*11:01:01	B*35:01:01	C*07:02:01	WT	WT
EC 002	1	A*01:01:01	B*15:01:01	C*03:03:01	∆32	WT
	2	A*24:02:01	B*57:01:01	C*06:02:01	WT	WT
EC 003	1	A*01:01:01	B*15:01:01	C*03:04:01	∆32	WT
	2	A*02:01:01	B*57:01:01	C*06:02:01	WT	WT
EC 004	1	A*02:01:01	B*15:01:01	C*03:03:01	WT	V64I
	2	A*11:01:01	B*35:01:01	C*04:01:01	WT	WT
LTNP 005	1	A*30:01:01	B*15:03:01	C*02:10:01	WT	V64I
	2	A*74:01:01	B*39:10:01	C*12:03:01	WT	WT
LTNP 006	1	A*02:01:01	B*13:02:01	C*02:02:02	∆32	V64I
	2	A*68:01:02	B*27:05:02	C*06:02:01	WT	WT
LTNP 007	1	A*02:01:01	B*15:10:01	C*03:04:02	WT	V64I
	2	A*74:01:01	B*44:07	C*04:01:01	WT	WT
LTNP 008	1	A*02:01:01	B*07:02:01	C*05:01:01	WT	V64I
	2	A*03:01:01	B*83:01	C*07:02:01	WT	WT
LTNP 009	1	A*11:01:01	B*51:01:01	C*15:02:01	WT	WT
	2	A*11:01:01	B*51:42	C*16:01:01	∆32	WT
LTNP 010	1	A*23:01:01	B*53:01:01	C*06:02:01	WT	V64I
	2	A*30:02:01	B*57:03:01	C*07:01:02	WT	V64I
LTNP 011	1	A*01:01:01	B*13:02:01	C*06:02:01	∆32	WT
	2	A*32:01:01	B*13:02:01	C*07:04:01	WT	WT

HLA-subtypes are divided into: Protecting (italics type), neutral (normal type), and susceptible (bold type) according to literature classification. Protective HLA-alleles: A*0202, A*0205, A*0214, A*2402, A*25, A*3201, A*6802, B*13, B*1302, B*14/Cw*0802, B*27, B*2705, B*52, B*57, B*5701, B*5703 (in Africans), C*8, C*14, and DRBl*01^10,11,52–57^. High-risk HLA-alleles: A*1, A*2301, A*29, B*8, B*22, B*35, B*3502, Cw*04, Cw*07, C*16, and DR3^10–12,52,54,57,58^. Neutral HLA-alleles: HLA-A2^8^. Common chemokine receptor alleles associated with slow progression: ∆32 and V64I. Wildtype (WT).

### Identification of rare genetic variants in ECs and LTNPs by whole exome sequencing (WES)

**Figure 3:**
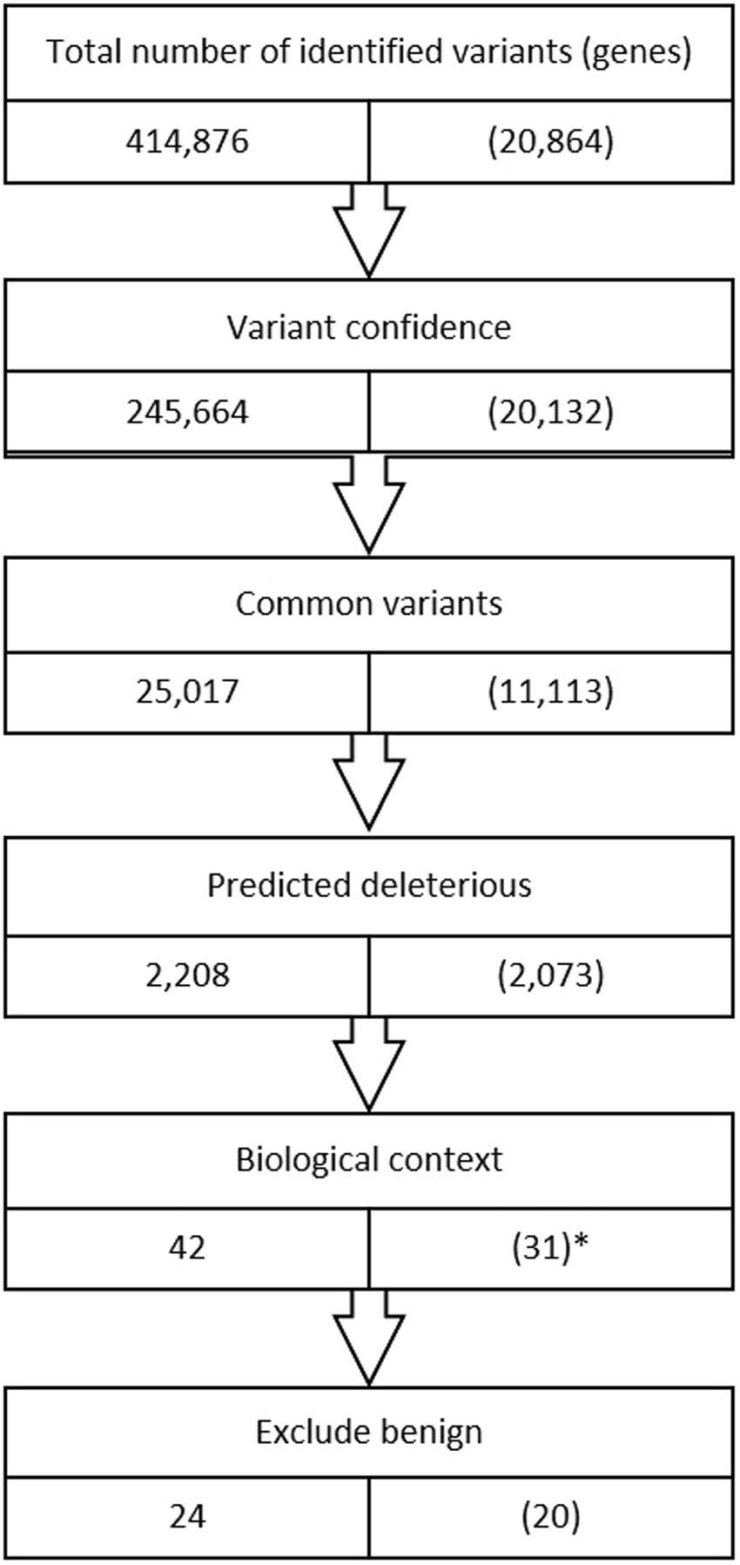
WES filtering diagram. Flowchart for whole exome sequencing (WES) filtering strategies according to quality, rareness, deleteriousness, and biological filters. Variant confidence: Keep only variants Outside top 1% most exonically variable genes and 5% most exonically variable 100 bases, quality >30, read depth 25, allele fraction >40. Common variants: Exclude variants >0.5% frequency in any reference genome. Predicted deleterious: Keep only >2 bases into intron; pathogenic or likely pathogenic variants according to ACMG guidelines or listed in HGMD; or frameshift, indels, and start/stop codon changes; missense unless predicted tolerated by SIFT or PolyPhen-2, CADD >20, splice site loss 2 bases into intron or predicted by MaxEntScan. Biological context: Keep only genes associated with innate sensing or HIV according to literature or IVA., Exclude benign variants with CADD <20 (no exceptions), CADD < MSC, tolerated by SIFT (no exceptions), and variants failing BAM file quality check. Known variants in HIV co-receptors (CCR2 and CCR5) were identified by a manual search. The filtering resulted in a total of 24 variants localized in 20 different genes among the 11 patients. *For variants annotated to two genes, only genes associated with HIV or innate sensing are counted. For further details, see supplementary text.

WES was performed with the aim of exploring further the genetic basis of the slow progressing EC and LTNP phenotypes. By this method, a total of 414,876 genetic variants were identified among the eleven ECs and LTNPs. In order to identify variants with possible impact on disease progression, the variants were filtered by Ingenuity Variant Analysis (IVA) software using a biologically relevant filter (Fig. 3 and relevant genes as shown Supplementary Table 3). Since the EC and LTNP phenotypes combined represent less than 1% of the HIV population, it can be assumed that less than 1% of the reference genomes would originate from an individual who would turn out to be ECs or LTNPs, if they had they been HIV infected. Thus, when filtering for rare variants represented in less than 0.5% of the reference genomes, such variants are unlikely to be present in a typical non-controlling HIV patient. During the filtering process, variants were already assessed based on CADD score with MSC, SIFT, and to some degree PolyPhen-2 (see supplementary text for details). To further evaluate which variants with high likely-hood could contribute to slow HIV disease progression, we assessed the PolyPhen-2 variant function prediction together with the likely-hood for the genes to be intolerant to variants using three different in silico scores: The Residual Variation Intolerance Score (RVIS), the Exome Aggregation Consortium (ExAC) missense Z score, and the ExAC LoF pLI score (Table 3 and Supplementary Table 4). We combined the result from the various bioinformatics tools. Based on this, we predicted the following variant-affected genes to be phenotype contributing in the carrier patients: *TAB2* in EC 004, *PIK3C2B* in LTNP 005, *MAP1A* together with *PIK3R5* in LTNP 006, *FGD6* in LTNP 007, *PRKDC* in LTNP 008, the combination of two variants in *FN*1 in LTNP 009, *PRKDC* in LTNP 010, and *PRKCA* in LTNP 011. On the other hand, less influence is suspected from variants in the following genes: *MMP9*, *EGF*, *CMA1*, *IRAK2*, *NOD2*, *MED6*, *PRIK3R6*, *SLX4*, and *CCNT1*.

**Table 3:** Rare variants identified in EC and LTNP individuals.

Patient number	Gene symbol	Gene function	Gene region	Protein variant	Transcript variant	GnomAD Freq.	CADD	MSC	PP2
EC 001									
EC 002 A*24, B*57, ∆32									
EC 003 B*57, ∆32									
EC 004	*DDOST*	Glycosylation of ENV on HIV, infectivity	Exonic	p.T400I	c.1199 C > T	0.114	32.0	3.3	Pr.D
	*TAB2*	Bacterial sensing	Exonic	p.R576H	c.1727G > A	0.000	33.0	<1	Pr.D
	*MMP9*	Endocytosis of HIV-1	Exonic	p.R652W	c.1954C > T	0.001	24.1	3.3	B
LTNP 005	*PIK3C2B*	HIV nuclear import	Exonic	p.E1169K	c.3505 G > A	0.008	27.9	3.3	B
	*FRK*	HIV binding and import	Exonic	p.V362A	c.1085 T > C	0.021	27.6	3.3	Pr.D
LTNP 006 B*13,	*EGF*	Increases activation of HIV LTR	Exonic	p.Y903C	c.2708 A > G	0.001	26.2	3.3	Pr.D
B*27, ∆32	*MAP1A*	HIV nuclear import	Exonic	p.P2690S	c.8068 C > T	0.145	25.6	3.3	Pr.D
	*PIK3R5*	HIV nuclear import	Exonic	p.L90V	c.268 C > G		26.9	3.3	Pr.D
LTNP 007	*FGD6*	HIV inward trafficking	Exonic	p.K46Q	c.136 A > C	0.078	22.2	3.3	Po.D
	*CMA1*	Chymotryptic serine proteinase	Exonic	p.R33C	c.97 C > T	0.025	24.6	3.3	B
LTNP 008	*FN1*	CD4-dependent infectivity	Exonic	p.R592H	c.1775G > A	0.292	29.5	28.4	Po.D
	*LRRFIP1*	Innate sensor	Exonic	p.M151V	c.451 A > G	0.002	26.3	3.3	Pr.D
	*IRAK2*	Bacterial sensing	Exonic	p.P421L	c.1262 C > T	0.004	31.0	3.3	Pr.D
	*PRKDC*	Interacts with Tat	Exonic	p.L1707Q	c.5120 T > A	0.217	25.4	3.3	Pr.D
	*MED6*	Interacts with Tat	Exonic	p.R183G	c.547 C > G	0.000	25.4	15.8	Po.D
	*NOD2*	Bacterial sensing	Exonic	p.T189M	c.566 C > T	0.230	26.0	<1	Pr.D
LTNP 009 ∆32	*FN1*	CD4-dependent infectivity	Exonic	p.R2425H	c.7274 G > A	0.036	34.0	28.4	B
	*FN1*	CD4-dependent infectivity	Exonic	p.P2016L	c.6047 C > T	0.032	34.0	28.4	B
	*PIK3R6*	HIV nuclear import	SSL	c.2109-1 G > A	0.096	25,5	3.3		
LTNP 010 B*57	*DDOST*	Glycosylation of ENV on HIV, infectivity	Exonic	p.V255I	c.763 G > A	0.017	23.8	3.3	B
	*SLX4*	Vpr-mediated G2/M arrest and chronic IFN production	Exonic	p.R1479*	c.4435 C > T	0.002	37.0	<1	
	*PRKDC*	Interacts with Tat	Exonic	p.L3073F	c.9217 C > T	0.095	21.2	3.3	Po.D
LTNP 011	*CCNT1*	Interacts with Tat	Exonic	p.R572H	c.1715G > A	0.060	27.1	3.3	Pr.D
B*13, B*13, ∆32	*PRKCA*	HIV nuclear import	Exonic	p.V344L	c.1030 G > T	0.005	24.9	3.3	B

Variants are shown according to individual patients. No variants were identified in EC 001, EC 002, and EC 003. Full gene names and further information can be found in Supplementary Table 4. In variant annotations, stop codons are marked with*. Mutation Significance Cutoff (MSC); Combined Annotation Dependent Depletion (CADD) score; splice cite loss (SSL); frequency (Freq.); PolyPhen-2 score (PP2); Probably damaging (Pr.D); Possibly damaging (Po.D); Benign (B).

**Figure 4:**
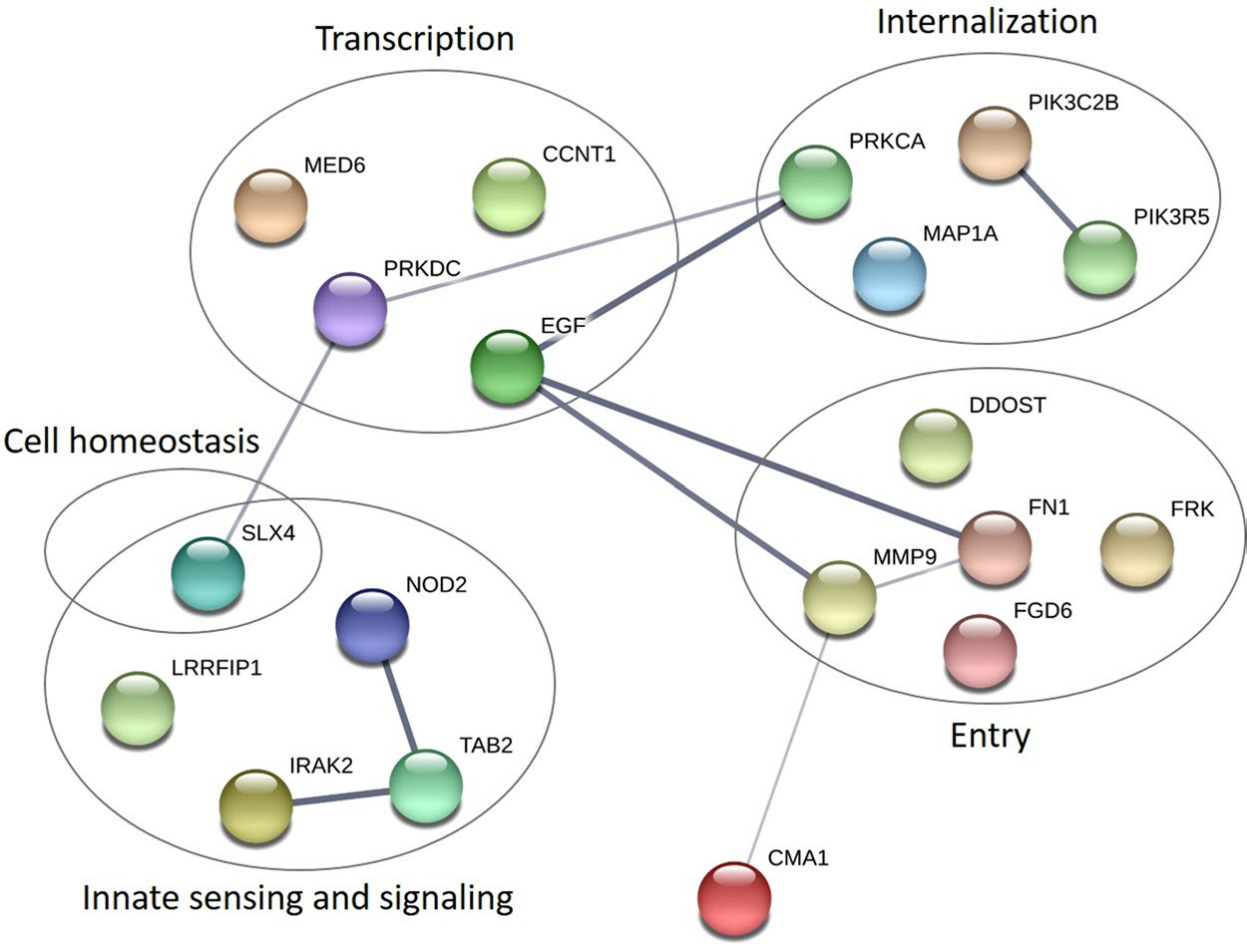
STRING association network for proteins affected by genetic variants within the HIV EC and LTNP patient cohort. Protein-protein interaction network for proteins encoded by genes with variants in the EC and LTNP cohort. *PIK3R6* is not a part of the network due to absence from the database. Each circle represents a gene/protein affected by at least one variant in the cohort. Thickness of gray lines represents strength of data supporting association, i.e. proteins jointly contribute to a shared function. Interactions are made with STRING version 10.5 based on data from genomic context predictions, high-throughput experiments, co-expressions, automated text-mining, and database search. Circles are drawn around proteins involved in common pathways or with similar biological functions. The PPI enrichment p-value for the number of identified interactions (10) compared to expected (5) in a group of 19 proteins was 0.0492, thus significantly more than expected, with a minimum interaction score a 0.4.

Following the filtering process, the number of relevant variants was reduced to 24, localized in a total of 20 different genes. An overview of identified variants in each individual is shown in Table 3. Further information on each variant and complete gene names are listed in Supplementary Table 4 (only abbreviations will be used in the following text). No variants were found in EC 001, EC 002, and EC 003 after the filtering process. Interestingly, both EC 002 and EC 003 harbored the protective *CCR5*-∆32 allele as well as the HLA-B*57 allele, the latter of which is one of the HLA alleles most highly associated with slow progression^19^. All identified rare variants were heterozygous and unique. However, different variants were localized within the same gene, both within one individual (*FN1*), hence potentially compound heterozygous, and between different individuals (*FN1*, *PRKDC*, and *DDOST*). Importantly, many of the genes containing variants have similar functions or are part of the same signaling pathway networks, suggesting a functional importance. To illustrate this point, all affected genes in the cohort are drawn in a STRING protein-protein network based on close functional associations or physical interactions (Fig. 4). The network had significantly (p = 0.0492) more interactions than expected (10 found versus 5 expected) for a random set of 19 different proteins (*PIK3R6* was not a part of the analysis since it was missing in the STRING database). This enrichment suggests some degree of biological connections between the genes carrying variants in these slow progressors.

#### Variants affecting HIV entry and trafficking

Three variants were identified in *FN1*, a gene affecting the CD4-dependent infectivity of HIV^20^. Five different variants were identified, all in different genes encoding proteins with a suggested role in nuclear import of HIV: *PIK3C2B*, *PIK3R5*, *PIK3R6*^21^, *MAP1A*, and *PRKCA*^22^. Furthermore, three variants were found in genes encoding proteins responsible for other parts of HIV trafficking: *FGD6* is a gene essential for HIV inward trafficking^23^, *MMP9* is involved in HIV-1 endocytosis^24^, and *FRK* has been attributed a role in DC immunoreceptor-mediated endosomal uptake triggered by HIV-1^25^. Moreover, two variants were identified in *DDOST*, which encodes a component of the oligosaccharyl transferase complex, which may be important for HIV Env glycosylation^26^, thus HIV import. Altogether, thirteen variants identified in the slow progressing cohort were found to influence HIV infectivity or inward trafficking.

#### Variants affecting innate sensing and inflammatory responses

Several genes encoding molecules with impact on innate sensing pathways and downstream IFN and pro-inflammatory cytokine production were affected: One variant was found in *LRRIF1P*, which is a dsRNA and dsDNA sensor responsible for IFN production^27^. Furthermore, one variant was identified in *IRAK2* and *TAB2*, which are both important for microbial sensing downstream of IL-1Rs/TLRs leading to NF-κB activation^28,29^. *TAB2* also signals downstream of *NOD2*, which also carried one variant. *NOD2* is an intracellular peptidoglycan sensor leading to inflammasome activation and IL-1 production after bacterial sensing^30^ and activation of the NF-κB pathway^31^. Additionally, one variant in *SLX4* was identified. *SLX4* is required for Vpr-mediated G2/M cell cycle arrest, and the SLX4 complex subunits are also responsible for suppressing spontaneous IFN production^32^. Overall, five variants with potential influence on the level of inflammation were present in the slow progressing cohort.

#### Variants affecting HIV transcription

One variant was identified in *EGF*, a protein promoting binding to the HIV LTR and facilitating transcription^33^. Similarly, several variants were found in genes affecting transcription induced by Tat, including *MED6*^34^, two variants in *PRKDC*^35^, and one variant in *CCNT1*^23,36,37^. Thus, five variants with potential impact on HIV transcription and replication were identified in the cohort.

#### Variants with diverse effects on HIV infection

Finally, one variant was identified in the *CMA1* gene previously found to be important in macaques controlling SIV^38^.

### Variants identified in a random control cohort of eleven herpes simplex encephalitis (HSE) patients

In order to verify that the identified gene variants in the slow progressing HIV patients were unique for this phenotype, WES analysis using identical selection and filtering tools was performed on a cohort of eleven HSE patients, which already had their genomes sequenced for research and diagnostic purposes^39^. A total of 18 variants in 14 genes were identified in this HSE cohort (Supplementary Table 5). Of these, three were pairs of similar variants, one pair in *PIK3C2G* and two pairs in *RNASEL*15, resulting in 15 unique variants. Three of the eleven HSE patients had no variants identified. As expected, most variants identified in the HSE cohort were linked to the TLR3 pathway known to increase the susceptibility to HSE^39–41^. Furthermore, several variants were identified in genes encoding viral restriction factors, such as RNaseL (*RNASEL*). Altogether, among the possible 523 different genes (according to the biological filter used in the WES analysis, Supplementary Table 3), only four genes were harboring variants in both the HIV slow progressor cohort and the HSE cohort: namely *PIK3R6*, *NOD2*, *IRAK2*, and *CCNT1*. None of the variants were identical between the two cohorts. Hence, we conclude that despite this minor overlap, all variants identified as well as the majority of the affected genes involved were unique and specific to the slow progressing HIV cohort. A STRING association network was also created for the genes affected by variants in the HSE control cohort (Supplementary Fig. 1). Contrary to the HIV EC/LTNP STRING network, the HSE STRING network did not display an enrichment of interactions: Four interactions were created between the 13 proteins, compared to two interactions expected, resulting in a non-significant protein-protein interactions (PPI) enrichment p-value of 0.0725.

### Innate immune responses

**Figure 5:**
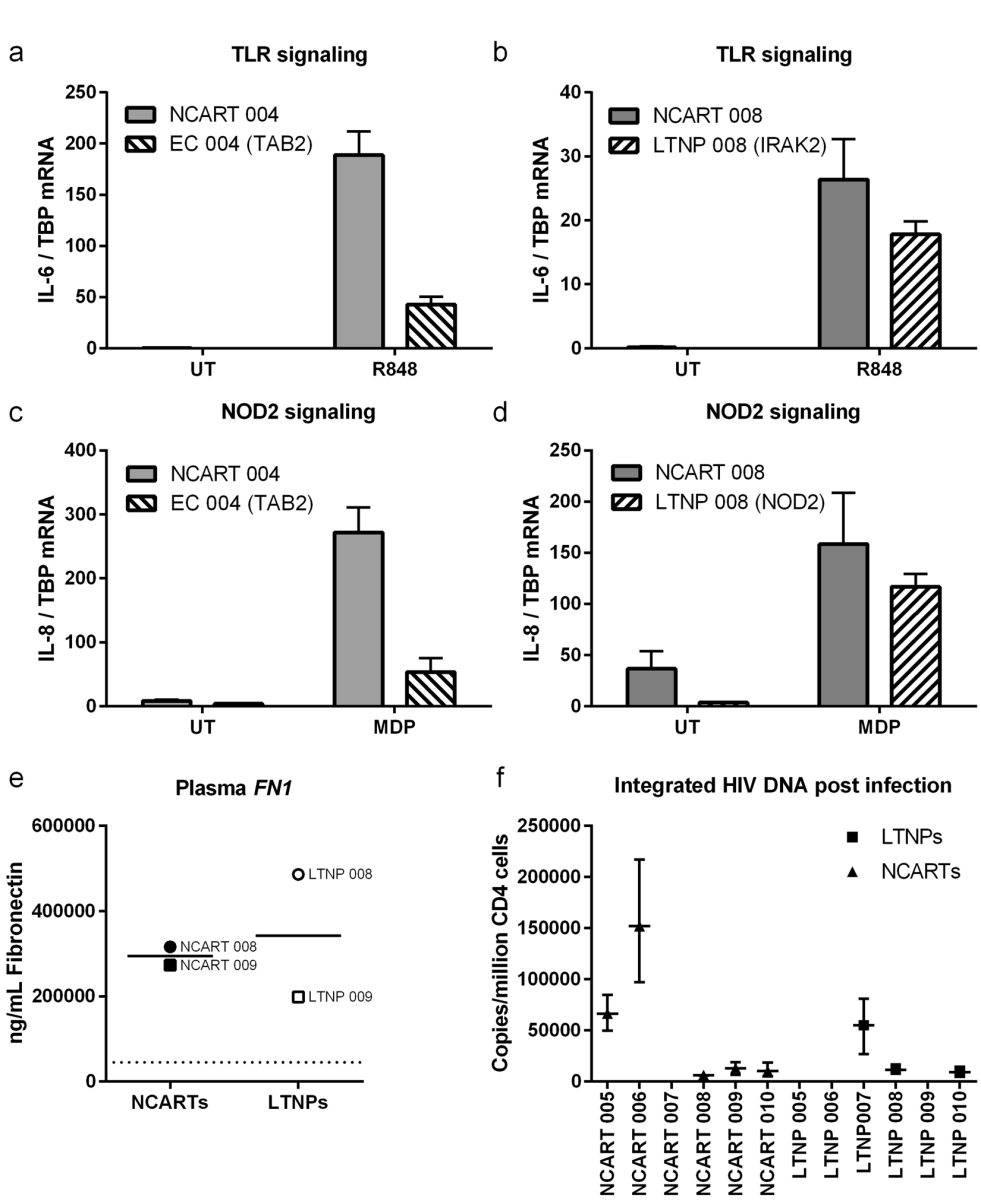
Functional assessment of variants affecting the TLR and NOD2 pathways, fibronectin plasma levels, and HIV replication upstream integration. (**a**,**b**) Patients with variants affecting the TLR sensing pathways (*IRAK2* and *TAB2* variants) and their a priori matched controls were stimulated with the TLR7/8 ligand Resiquimod (R848) (1 µg/mL) for 6 hrs followed by IL-6 qPCR. (**c**,**d**) Patients with variants affecting the NOD2 sensing pathways (*NOD2* and *TAB2* variants) and their a priori matched controls were stimulated with the NOD2 agonist muramyl dipeptide (MDP) (1 µg/mL) for 6 hrs followed by IL-8 qPCR. Means of triplicates with standard derivation are shown. (**e**) Measurements of plasma fibronectin levels in patients with variants in *FN1* encoding fibronectin and in age- and gender-matched controls. Means are shown with each dot representing mean values from technical duplicates based on ELISA. Dotted line represents detection limit. (**f**) Integrated HIV DNA after infection with the HIV strain HXB2 at MOI 0.1 with endogenous levels of integrated HIV DNA subtracted. Integrated HIV DNA was measured in LTNPs harbouring variants potentially affecting HIV inward trafficking and integration (*PIK3C2B*, *FRK*, *MAP1A*, *PIK3R5*, *FGD6*, *FN1*, *PIK3R6*, and *DDOST*) and in age- and gender-matched controls. Error bars represent min and max values from technical replicates. Non-controller on ART (NCART); long-term non-progressor (LTNP); elite controller (EC); untreated (UT).

In order to investigate innate immune responses, we stimulated patient PBMCs with dsDNA from herring testes (htDNA) and Sendai virus (SeV) and measured pro-inflammatory and antiviral immune responses. The level of CXCL10, IFNβ, TNFα, and IL-6 mRNA induction were determined by qPCR. We observed a tendency towards a reduction in both IFNβ and CXCL10 after DNA transfection in the EC group compared to NCARTs, but collectively there were no significant differences in induction of cytokines between the NCARTs and ECs or LTNPs (Supplementary Fig. 2). However, when examining the functional effect of single variants in individual patients and their matched controls using a TLR7/8 agonist (R847) and a NOD2 agonist (MDP), remarkable differences were observed (Fig. 5). EC 004 showed markedly reduced IL-6 and IL-8 responses compared to the matched control with regards to TLR7/8 and NOD2 activation, respectively (Fig. 5a,c). This reduced pro-inflammatory response may be attributed to a variant in the *TAB2* gene acting downstream of both TLR and NOD2 sensing. Likewise, trends towards reduced IL-6 and IL-8 production after TLR7/8 and NOD2 activation, respectively, were seen for LTNP 008 compared to the matched control (Fig. 5b,d). LTNP 008 harbors a variant in both *IRAK2* downstream of TLRs and a variant in *NOD2*, which could explain this slight reduction in pro-inflammatory responses.

### Functional validation of variants affecting HIV infection, -inward trafficking, and -nuclear import in LTNPs

Three variants within the fibronectin encoding gene *FN1* were identified: a R592H variant in LTNP 008 and the two variants R2425H and P2016L in LTNP 009. Fibronectin is an important extracellular matrix protein affecting HIV infectivity^20^, and is also found in a soluble form in the plasma. These missense variants may affect the distribution of different splice variants, proteolytic cleavage of the protein or protein stability, and could therefore potentially have influence on the amount of fibronectin in plasma. We therefore investigated the plasma level of fibronectin in LTNP 008 and LTNP 009 compared to age- and gender-matched controls. LTNP 008 with the single R592H-*FN1* variant had normal fibronectin plasma level, whereas LTNP 009 with the double variant R2425H-*FN1* and P2016L-*FN1* displayed plasma fibronectin levels in the lower range of normal and reduced levels compared to the matched control (Fig. 5e).

Next, we functionally examined variants potentially affecting nuclear import: *PIK3C2B* and *FRK* (LTNP 005), *PIK3R5* and *MAP1A* (LTNP 006), and *PIK3R6* (LTNP 009); HIV inward trafficking: *FGD6* (LTNP 007); as well as HIV infectivity: *FN1* (LTNP 008 and 009) and *DDOST* (LTNP 010). (*PRKCA* in LTNP 011, which potentially also affects HIV nuclear import was not investigated due to lack of patient material). All these variants may potentially affect the HIV replication cycle upstream of HIV integration, but before synthesis of HIV particles, and independently of their *CCR5* and HLA alleles. We therefore assessed the different variants by infecting patient CD4 T cells with an X4-HIV strain, measured the level of integrated HIV DNA, and estimated the endogenous level of HIV DNA by subtracting the level in uninfected samples. Interestingly, the level of newly integrated HIV DNA was only detectable in three (LTNP 007, LTNP 008, and LTNP 010) of six LTNPs, whereas it could be detected in five (all except NCART 007) of six matched NCARTs (Fig. 5f). These data may suggest, although they do not prove, that the identified variants, especially in LTNP 005, LTNP 006, and LTNP 009, may negatively affect the HIV replication cycle upstream of viral integration.

## Discussion

Despite the relatively limited size of our cohort, we here demonstrate a significant difference in total HIV DNA in CD4+ T cells in both ECs and LTNPs as compared to NCARTs. This finding is consistent with findings by Graf *et al*., who demonstrated a similar trend, whereas our study is the first to demonstrate a significant difference^18^. The findings of a smaller HIV reservoir in the EC and LTNP individuals confirm correct patient selection and grouping of the phenotypes. Interestingly, those who had the lowest HIV DNA level also seem to be controlling the infection for the longest time span. A possible explanation of the small reservoir size in the ECs and LTNPs may be a result of variants in genes potentially influencing total HIV DNA generation during HIV replication, viral inward trafficking, and integration. Indeed, all LTNPs but only one of four ECs (EC 004) harbored variants in genes affecting HIV infectivity and nuclear import. In fact, newly integrated HIV DNA was not detected in LTNP 005, LTNP 006, and LTNP 009 after 24hrs infection with an X4-HIV strain.

The major finding of the present study is the identification of several rare genetic variants, particularly among LTNPs, that we suggest may contribute to their favorable disease course. These rare variants have not previously been identified in other genetic studies on ECs and LTNPs, which have focused on common variants or genes affected in a large fraction of ECs or LTNPs. With the present approach, we have examined the exome of each slow progressor individual comprehensively. We searched for variants that were less frequent than the EC and LTNP phenotypes among HIV-infected individuals. Extremely rare variants with different localization within a gene or pathway may result in a similar more common phenotype as described for certain rare primary immunodeficiencies^42^. Through specific filtering approaches, we discovered several novel interesting variants that are part of innate immune sensing pathways or involved in the HIV replication cycle, which we suggest may contribute to the genetically heterogeneous EC and LTNP phenotypes of these patients.

Remarkably, out of the four ECs, only one carried variants after the filtering process, whereas all seven LTNPs carried rare variants. Both EC 002 and EC 003 carried both *HLA*-B*57 and *CCR5*-∆32, indicating that the combination of these alleles may be sufficient to cause an EC phenotype. On the other hand, a combination of rare variants seems to result in the less controlling, but still favorable, LTNP phenotype. These findings may suggest a different genetic background and pathogenesis between the EC versus the LTNP phenotypes. However, given the limited number of patients eligible for inclusion in the present study, this remains a hypothesis that needs to be further investigated in future studies on HIV EC and LTNP patients. Another explanation for the lack of variants in the three ECs might be that we only examined exons and the beginning of splice sites. Therefore, important variations in introns, promoter-, and regulatory sequences may have gone unnoticed in the present study.

An interesting finding was three variants in two different LTNP patients in the *FN1* gene affecting CD4-dependet HIV infectivity. Thus, this mechanism and particularly *FN1* may be important for the LTNP phenotype. Interestingly, when examining the functional effect of *FN1* variants on the expression level of soluble fibronectin in plasma, we found that LTNP 009 with the two variants in *FN1* and with high CADD scores at 34, displayed low plasma fibronectin levels compared to the age- and gender-matched control. On the other hand, LTNP 008 with only one variant in *FN1*, with a CADD score just above the MSC, showed high fibronectin plasma levels. However, fibronectin levels in plasma have high inter-individual variance. Furthermore, the three variants in *FN1* were all missenses variants and might not all affect the expression level, but rather affect the multimeric formation in the extracellular matrix or affect binding to HIV or other proteins. Therefore, to fully understand the role of fibronectin in HIV disease progression further studies in relevant tissue needs to be conducted.

Another important discovery from the present study was the finding of of nine additional variants in genes described to affect HIV uptake and intracellular trafficking. To our knowledge, this is the first time HIV-trafficking has been associated with the slow progressing phenotypes, particularly in LTNPs. To assess these variants, we examined the amount of newly integrated HIV DNA post a single round of *in vitro* X4-HIV infection. Interestingly, newly integrated HIV DNA was only detectable in three out of the six investigated LTNPs compared to the majority (five out of six) matched NCARTs. Thus, the *FRK*, *PIK3C2B*, *PIK3R5*, *MAP1A*, and *PIK3R6* genes present in LTNP 005, LNTP 006, and LTNP 009, which are suggested to play a role in HIV nuclear import, and all with *in vitro* newly integrated HIV DNA below detection level, represent highly interesting candidate genes for further studies on the mechanisms underlying HIV slow progression. However, these findings need to be interpreted with precaution, since the variability in the assay is quite high due to the low copy numbers detected. Furthermore, these variants may not be solely responsible for the slow progressor phenotype but rather contributing, thus functional changes may be expected to be somewhat modest.

In addition to the variants affecting the first part of HIV replication cycle, we also found four variants in genes affecting HIV transcription; specifically linked to Tat and LTR-mediated transcription.

Innate immune sensing pathways were found to be affected by five variants. A decrease in type I IFN and pro-inflammatory responses due to these variants may contribute to a generally lower level of chronic immune activation as previously observed in studies on primates controlling SIV^16,43^. By examining the functional consequences of the identified variants in terms of immune responses to various ligands, we observed a tendency towards reduced IFNβ and CXCL10 responses to DNA, although these findings were not significant and would therefore be relevant to investigate in a larger cohort of patients. However, since the variants identified here belongs to different cellular signaling pathways, their responses to stimulation would not be expected to provide a uniform picture, since it was impossible to select a ligand or agonist that would be able to reflect the diversity of pathways involved. Interestingly, a more selective functional analysis of the *TAB2* variant in EC 004, which was predicted highly deleterious, and the *IRAK2* and *NOD2* variants in LTNP 008, which were both predicted less deleterious, confirmed our in silico predictions (Supplementary Table 2). Downstream of both TLR7/8 and NOD2 sensing the deleterious *TAB2* variant resulted in markedly reduced pro-inflammatory cytokine response to these pathways. On the other hand, the *IRAK2* (downstream of TLRs) and *NOD2* variants resulted in only slightly reduced pro-inflammatory responses to these pathways, although the combined effect of the variants might have some biological penetrance. This indicates that reduced chronic immune activation after sensing of microbial PAMPs may contribute to the slow disease progression in certain slow progressors, such as EC 004.

To limit the number of candidate variants and to minimize false-positive variants with no impact on disease pathogenesis we used relatively strict filtering criteria. To challenge the hypothesis or assumption that the identified variants in the EC and LTNP cohort were specifically linked to HIV pathogenesis, we performed the exact same WES analysis with similar filtering criteria in a random control cohort of eleven HSE patients. Based on these combined analyses, only four genes were found to harbor variants in both cohorts. However, a variant increasing the potential of being an EC or LTNP cannot be excluded to not simultaneously increase the risk of acquiring HSE. Thus, while an impaired type I IFN response is detrimental for overcoming severe HSV infection, this reduced chronic IFN production may be beneficial to avoid chronic immune activation during HIV infection. The variants in *NOD2* found in the HSE patient as well as in EC 004 are relatively common in Caucasians, although rare in the GnomAD reference genomes, and thus might represent false positive variants. Concerning the *IRAK2* variant in LTNP 008, this variant is located in the kinase domain of the molecule, hence probably affecting the protein function to some degree. This is supported by the functional data demonstrating a trend towards reduced cytokine responses in LTNP 008 downstream of TLR signaling through *IRAK2*. Therefore, the combined effect of an impaired IRAK2 protein and an impaired NOD2 protein may reduce chronic inflammation, which possibly together with other factors may explain the rare slow progressing phenotype in this patient.

Taken together, the rare variants identified in the EC and LTNP cohort are most likely contributing to their slow progressing phenotype, possibly together with other rare variants as well as the common HLA and *CCR5* alleles. Data might be interpreted to suggest a role for different variants affecting various pathways involved in immune sensing and HIV replication in LTNPs, whereas the previously identified protective *CCR5-* and HLA-alleles may be a major determinant for the “pure” EC phenotype.

It should be taken into consideration that the bioinformatics software utilized in the analysis has been developed primarily to identify monogenic inherited deleterious variants and are therefore not ideal to identify variants contributing to an advantageous phenotype, as is the case in the present study. Consequently, the validity of variants cannot solely be based on WES analysis through *in silico* tools, but rather represent important novel candidate genes and proteins for future functional studies in patient cells and *in vitro*.

An important piece of evidence to support the validity of the identified variants is the significant enrichments of interactions in the slow progressor cohort found by the STRING analysis, contrary to variants identified in a random HSE cohort of similar size, using the same filtering approach. This indicates that despite the diversity of the genes harboring variants in the EC and LTNP cohort, these genes may encode proteins with some degree of interaction and common mechanisms or functions, ultimately resulting in a common clinical slow progressor phenotype. The different pathways and mechanisms found to be altered in the EC and LTNP group should therefore be investigated further for their role in slow progression and may potentially lead to discovery of new drug targets for supplementary HIV treatment.

Finally, despite the strict filtering criteria, we identified four individuals with rare variants and without the common protecting *CCR5*-∆32 allele nor any protective HLA alleles. On this basis, we suggest that the gene variants in EC 004 (*TAB2*), LTNP 005 (*PIK3C2B*), LTNP 007 (*FGD6*), and LTNP 008 (*PRKDC*) may be of particular interest and worth exploring further as contributors to the slow progressing phenotype. *PRKDC* may be of particular interest, since it was also found to be affected in both LTNP 008 and LTNP 010, who both had low, although detectable, levels of integrated HIV DNA after infection.

Importantly, the combination of two rare variants in *FN1* in LTNP 009, who presented with low plasma fibronectin levels, and the fact that this gene was also affected in another patient (LTNP 008), suggest that *FN1* may be a major factor in the LTNP phenotype. Further studies are required to establish the role of FN1 in HIV disease progression, since this was not possible in the present study due to the extracellular matrix localization of this protein. Lastly, it is worth noticing that LTNP 006 had two variants potentially affecting HIV nuclear import by the *PIK3R5* and *MAP1A* genes and had undeletable levels of newly integrated HIV DNA post infection, which cannot be attributed to the more common protective alleles in this LTNP. Hence, the role of HIV nuclear import in HIV disease progression should be further investigated.

## Conclusion

In the present study, we identified several interesting variants in genes encoding molecules involved in HIV entry and inward trafficking, HIV transcription, cell homeostasis, sensing, and inflammation. The picture is complex and there appears to be no single unifying mechanism common to all HIV ECs and LTNPs that may explain their favorable phenotype consisting of slow HIV disease progression. An important and unexpected finding was the presence of rare genetic variants in all LTNPs examined but only in one of four ECs, which may suggest a different genetic background and pathogenesis distinguishing these two different HIV disease phenotypes. We suggest that the precise functional differences between ECs, LTNPs, and controls should be further studied in larger cohorts of HIV patients. Even though many of the variants affect different genes, a functionality STRING network analysis revealed a significantly higher degree of relatedness between these genes than what would be expected in a random sample. Based on our data, we suggest that one or more rare genetic variants present in different genes, but in functionally related pathways, may be a contributing factor to the genetically heterogeneous LTNP phenotype, which may be distinguishable from the genetic basis for the EC phenotype. These molecules and the immunological and cell biological mechanisms, in which they are involved, may serve as candidate targets for further studies on HIV pathogenesis and disease progression and may also indicate potential novel drug targets. Hence, understanding the mechanisms underlying the EC and LTNP phenotypes may pave the way for new treatment approaches, such as immunomodulatory therapy to treat HIV-related residual inflammation and support ART non-responders in regaining normal CD4 count.

## Methods

### Study population and design

We conducted a cross-sectional study enrolling HIV ECs and LTNPs for WES analysis and investigation of their innate immune response. After inclusion, a priori to stimulation experiments, ECs and LTNPs were age- and gender-matched to HIV NCARTs (Supplementary Table 1). The study participants were enrolled at the Departments of Infectious Diseases from four Danish hospitals: Aarhus University Hospital, Copenhagen University Hospital, Hvidovre Hospital, and Aalborg University Hospital. Study participants were identified based on data from The Danish HIV Cohort study^44^. Inclusion criteria for ECs: Age >18 years, HIV diagnosed for at least 5 years, at least 3 VL measurements with at least one year between first and last measurement, and not more than one CD4 T cell count <500 cells/µL. Exclusion criteria: Anti-retroviral treatment, AIDS-defining events, VL measurement ≥1000 copies/mL, two consecutive VL measurements >400 copies/mL, and >20% of VL measurements above detection limit. Inclusion criteria for LTNPs: Age >18 years, HIV diagnosed for at least 5 years, at least 3 VL measurements with at least one year between first and last measurement, and CD4 count >350 cells/μL by two consecutive measurements. Exclusion criteria: Anti-retroviral treatment, AIDS-defining events, two consecutive VL measurements ≥2000 copies/mL, and >20% of VL measurements ≥2000 copies/μL. Inclusion criteria for NCARTs: Age >18 years, HIV diagnosed for at least 5 years, CD4 count should have been below 350 cells/µL leading to initiation of ART within the first 5 years of HIV diagnosis, and ART for ≥1year.

### Ethics

The Danish National Committee on Health Research Ethics (1-10-72-369-14) and the Danish Data Protection Agency (Journal number 2007-58-0010) approved the project. WES was only permitted for the EC and LTNP group and not the NCART group due to potential risk of unintentionally identifying non-HIV-related disease variants. All patients provided informed written consent prior to study inclusion. All protocols were approved and methods carried out were in accordance with relevant guidelines and regulations. Some of the HIV patients included here have also been described in another study^45^.

For the data and WES analysis of the HSE cohort utilized for comparison, that study was approved by the Danish National Committee on Health Research Ethics (1-10-72-275-15) and previously published^39^.

### PBMC purification and stimulation

Blood was collected in EDTA tubes and separated by ficoll (GE Healthcare) gradient in SepMate tubes (Stemcell Technologies). PBMCs were stored in liquid nitrogen. Frozen PBMCs were resuspended in complete RPMI: RPMI 1640 medium with L-glutamine supplemented with 10% heat inactivated (HI) FBS, 100 IU/ml penicillin, and 100 µg/ml streptomycin, all from Biowest. PBMCs where incubated overnight at 37 °C in 5% CO_2_ before stimulations with: 1 µg/mL Resiquimod (R848) (Invivogen), 1 µg/mL muramyl dipeptide (MDP) (InvivoGen), Sendai Virus (SeV) Cantell Strain (VR-907 from ATCC, Mannassas, VA, USA) (1:500 volume), or 2 µg/mL htDNA (Sigma Aldrich) transfection using Lipofectamine 3000. The Lipofectamine:DNA ratio was 1:1, and was diluted in OptiMem (Gibco by Life Technologies) following manufactures instructions. Lipofectamine 3000 without DNA was used as mock control. Complete RPMI was used as untreated (UT) control. Cell were lysed after 6 hrs using lysis buffer from High Pure RNA Isolation kit (Roche, Switzerland).

### CD4+ T cell and non-CD4+ cell isolation followed by DNA purification

CD4+ T cells were negatively isolated using the MACSxpress CD4 T cell Isolation Kit (Miltenyi). For non-CD4+ cell isolation, the column was removed from the magnet and washed with MACS buffer containing PBS (Biowest), EDTA (FLUKA), and BSA (Sigma) to release non-CD4+ cells. Cells were lysed in RLT+ buffer (Qiagen). DNA was extracted using AllPrep DNA/RNA Mini Kit (Qiagen).

### WES, variant calling and annotation

WES was performed on DNA from ECs and LTNPs employing Kapa HTP Library preparation and Nimblegen SeqCap EZ MedExome Plus kit and analyzed using Nextseq v2 chemiststry (2 × 150 bp). SNPs were called relative to hg19. Variant call files (VCF) were uploaded to Ingenuity Variant Analysis (IVA, Qiagen) and filtered (more details are provided in the Supplementary text). In brief, we sorted out variants that were calculated to be rare (present in <0.5% of the reference genomes), potentially damaging, and associated to HIV or innate sensing based on the literature or according to IVA (Fig. 3 and Supplementary Table 3). The Mutation Significance Cutoff (MSC) (with a 99% confidence Interval with HGMD Database Source)^46^ was calculated for the combined annotation dependent depletion (CADD) score^47^ to estimate impact of damaging variants. Variants with CADD lower than 20 or lower than the MSC for that given gene were assumed benign and excluded. Likewise, variants predicted to be tolerated by SIFT score were also excluded (Fig. 3). Variant filtering was verified by random sampling. All variants identified by IVA were manually confirmed or excluded after examination of BAM files using the UCSC genome browser.

The exact same filtering approach was used to identify genetic variants in an unrelated cohort of eleven herpes simplex encephalitis (HSE) patients, to estimate the specificity of the variants identified within the HIV cohort.

In order to estimate genetic intolerance, each variant-carrying gene was annotated with the Residual Variation Intolerance Score (RVIS) showing the percentage of more intolerant genes, according to data from the NHLBI-ESP6500 data set (Supplementary Table 4). Furthermore, the Exome Aggregation Consortium (ExAC) Z score for missense and synonymous variants was noted for each gene. A positive Z value indicates an intolerant gene, whereas a negative Z value indicates a gene with more variants than expected (Supplementary Table 4). The ExAC pLI score for loss-of-function (LoF) variants was also used to estimate a variant constraint index. A LoF pLI score ≤ 0.1 indicates a tolerant gene, whereas LoF pLI ≥0.9 indicates a completely intolerant gene (Supplementary Table 4).

More common variants in chemokine receptors known to be associated with slow HIV progression were identified by manual search in IVA.

### STRING analysis

All variant-carrying genes in the cohort were analyzed for associations regarding physical interactions or shared functionality using STRING (© STRING consortium 2017) Version 10.5^48^, creating an association network. A similar STRING association network was also created for the proteins affected by variants within the HSE cohort.

### HLA subtyping

HLA typing was performed by analyzing WES generated fastq files with HLA Explore v. 1.2.1 software (Omixon). Initially, fastq files were concatenated and filtered for the MHC region before performing HLA typing. The mean total number of best mapping reads was 19,300 (range 14,081-38,856) for all loci. At the low end, this resulted in a read depth of 15–70 in key exons. This generated three field typings for *HLA*-A, B, C, DPA1, DPB1, DQA1, DQB1, and DRB1.

### *CCR5*-∆32 PCR and agarose separation

In order to verify (in ECs and LTNPs) and identify (in NCARTs) the *CCR5*-WT and -∆32 allele, a PCR was run with a primer pair covering the deletion: (CCR5-D32-F: 5′CTTCATTACACCTGCAGCT3′ and CCR5-D32-R: 5′TGAAGATAAGCCTCACAGCC3′)^49^. PCR fragments of 196 bp for WT allele and 164 bp for the ∆32 allele were separated on a 2% agarose gel.

### RNA purification, cDNA synthesis and qPCR

RNA was purified by High Pure RNA Isolation kit (Roche). cDNA was synthesized from RNA using QuantiTect Reverse Transcription kit (QIAGEN). mRNA expression levels were determined by TaqMan qPCR using PerfeCTa® qPCR FastMix® II, ROX™ (Quantabio, USA) using the following primers and probes (gene, catalog nr., assay ID) all from Thermo Scientific: TBP (4331182, Hs00427620_m1), CXCL10 (4331182, Hs01124251_g1), TNFα (4331182, Hs01113624_g1), IFNB1 hCG28967 (4331182 Hs01077958_s1), IL-6 hCG38231 (4331182 Hs00985639_m1), and CXCL8 (IL-8) (4331182 Hs00174103_m1). All methods were performed following the manufacturers’ instructions.

### Total HIV DNA measurement by digital droplet (dd) PCR

The amount of total HIV DNA per million CD4+ T cells was measured by ddPCR as described previously^50,51^. After droplet generation, the PCR reaction was performed under the following conditions: 95 °C for 10 min followed by 45 cycles of 95 °C for 30 sec and 59 °C for 1 min, and finally 98 °C for 10 min. Following PCR amplification, the total HIV-1 DNA copy number was quantified in each sample using the QX200 Droplet Reader (Biorad).

### Integrated HIV DNA measurement post *in vitro* HIV infection

CD4 T cells were activated for 72 hrs in complete RPMI supplemented with 40 U/mL IL-2 (Gibco) and 1% PHA (Remel). PHA was removed and 300,000 cells were infected with the HXB2D HIV strain at MOI 0.1 after 2 hrs of resting. The cells were lysed after 24 hrs, allowing for a single round of infection, and total DNA was extracted. For each DNA sample, 350 ng of total DNA was loaded onto a 0.4% agarose gel and DNA was fractionated by electrophoresis in TAE buffer for 1.5 hrs at 110 V. The bands were visualized by post-staining of the gel with GelRed^TM^ (Biotium) according to the manufacturer’s protocol. The 20 kb high molecular weight (HMW) band were excised from the gel. DNA was extracted from the gel pieces using Qiaex II gel extraction kit (Qiagen) in accordance to the manufacturers’ protocol with the following modifications: the incubation time in QXI buffer + QIAEX II at 50 °C was extended to 20 minutes, and following incubation each sample was washed three times in QXI buffer. Integrated HIV-1 DNA was measured by digital droplet PCR (ddPCR) using the same reagents and methodology as described for measurement of total HIV-1 DNA.

### Plasma fibronectin measurement

Plasma levels of Fibronectin were measured using hFibronectin DuoSet ELISA (R&D systems Biotechne) following the manufacturer’s instructions. Each plasma sample was measured in technical duplicates.

### Statistics

Differences between NCARTs and ECs or LTNPs were calculated using non-parametric Mann-Whitney test on the unpaired samples, and Pearson correlations were used to determine associations. Two-tailed p values are stated. Statistics were calculated in Graphpad Prism 6.

## Electronic supplementary material

Supplementary Dataset

## Data Availability

All data are available upon request to the authors.
