# Supplementary material for: Whole Exome Sequencing of HIV-1 long-term non-progressors identifies rare variants in genes encoding innate immune sensors and signaling molecules
Source: Sci Rep. 2018 Oct 15;8:15253. doi: 10.1038/s41598-018-33481-0 (PMC6189090; doi:10.1038/s41598-018-33481-0)
Supplement: Supplementary file 1 — Supplementary Dataset [file 41598_2018_33481_MOESM1_ESM.pdf]

Supplementary material for the manuscript:

Whole Exome Sequencing of HIV-1 long-term non-progressors identifies rare variants in genes encoding innate immune sensors and signaling molecules

Authors

Sara Konstantin NISSEN, Mette CHRISTIANSEN, Marie HELLEBERG, Kathrine KJÆR, Sofie Eg JØRGENSEN, Jan GERSTOFT, Terese Lea KATZENSTEIN, Thomas BENFIELD, Gitte KRONBORG, Carsten Schade LARSEN, Alex LAURSEN, Gitte PEDERSEN, Martin Roelsgaard JAKOBSEN, Martin TOLSTRUP, and Trine Hyrup MOGENSEN.

The contents of this supplementary file is a detailed description of variant filtering methods, Supplementary Table 1-5, and Supplementary Figure 1-2.

Detailed description of variant filtering methods:

Starting with 414,876 variants spanning 20,864 genes, rare, deleterious, and biological relevant variants were identified using the following filter setting:

Variants kept based on sequencing confidence:

- With call quality at least 30
- AND with read depth at least 25
- AND with allele fraction at least 40 with genotype quality at least 30
- AND outside top 5.0% most exonically variable 100 base windows in healthy public genomes (1000 genomes)

- AND outside top 1.0% most exonically variable genes in healthy public genomes (1000 genomes).

Common variants excluded:

- With an allele frequency greater than or equal to 0.5% of the genomes in the 1000 genomes project
- OR greater than or equal to 0.5% of the NHLBI ESP exomes (all)
- OR greater than or equal to 0.5% of the AFC Frequency
- OR greater than or equal to 0.5% of the ExAC Frequency
- OR greater than or equal to 0.5% of the gnomAD Frequency.

Deleterious variants kept:

- (Up to 2 bases into intron) that are experimentally observed to be associated with a phenotype: Pathogenic, Possibly Pathogenic
- OR disease-associated according to HGMD
- OR frameshift, in-frame indel, or stop codon change
- OR missense unless predicted to be innocuous by SIFT or Polyphen-2
- OR predicted deleterious by having CADD score > 20
- OR predicted to disrupt splicing by MaxEntScan
- OR within 2 bases into intron.

Biological relevant variants kept (full biological filter gene list shown in Supplementary table 3):

- kept that are known or predicted to affect: HIV-1 Activation , HIV-1 Dimerization, HIV-1 Expression, HIV-1 Localization, HIV-1 attachment, HIV-1 binding, HIV-1 budding, HIV-1 co-localization, HIV-1 content, Quantity of HIV-1, HIV-1 deactivation, Inactivation of HIV-1, HIV-1 endocytosis, HIV-1 formation, HIV-1 fusion, HIV-1 immunoneutralization, Neutralization of HIV-1, or HIV-1 infection progression.

- OR associated to HIV, SIV, or innate immune sensing according to a literature search

Benign variants excluded:

- Predicted benign by having CADD score ≤ 20.
- OR having a CADD score ≤ MSC (with 99% conficence interval)
- OR tolerated SIFT score

Ingenuity Variant Analysis version 5.2.20180316

Content versions: CADD (v1.3), CentoMD (4.1), EVS (ESP6500SI-V2), Allele Frequency Community (2018-01-17), JASPAR (2013-11), Ingenuity Knowledge Base Snapshot Timestamp (2018-03-22 15:45:20.0), Ingenuity Knowledge Base (Pandora 180322.000), Vista Enhancer (2012-07), OMIM (May 26, 2017), gnomAD (2.0.1), Clinical Trials (Pandora 180322.000), BSIFT (2016-02-23), TCGA (2013-09-05), PolyPhen-2 (v2.2.2), 1000 Genome Frequency (phase3v5b), Clinvar (2018-01-03), DGV (2016-05-15), COSMIC (v83), ExAC (0.3.1), HGMD (2017.4), PhyloP (2009-11), DbSNP (150(2017-07-10)), TargetScan (6.2), SIFT4G (2016-02-23).

Supplementary figures and tables:

Supplementary Table 1: Individual characteristics for the study group of elite controllers (ECs) and long-term non-progressors (LTNPs), as well as the control group of non-controllers on ART (NCARTs).

**NCART nr**	**001**	**002**	**003**	**004**	**005**	**006**	**007**	**008**	**009**	**010**	**011**
Nadir CD4	266	150	100	20	180	10	180	33	40	149	40
ART-free years	2.89	0.085	1.18	1.01	1.42	0.11	2.51	0.05	0.06	0.13	0.08
Years with HIV	22.22	18.93	22.22	23.16	20.76	18.45	17.42	15.62	13.58	12.96	19.23
CD4 at inclusion	1000	1170	420	80	440	260	890	666	500	630	420
VL at inclusion	19	50	30	46	19	19	19	45	19	19	19
Age	69	54	72	52	48	77	48	44	66	53	57
Gender	M	M	M	M	F	M	F	F	M	F	M
Ethnicity	C	C	C	C	A	C	A	A	C	A	C
CCR5 genotype	WT/WT	WT/WT	Δ32/WT	WT/WT	WT/WT	WT/WT	WT/WT	WT/WT	WT/WT	WT/WT	Δ32/WT
**EC/LTNP nr**	**EC 001**	**EC 002**	**EC 003**	**EC 004**	**LTNP 005**	**LTNP 006**	**LTNP 007**	**LTNP 008**	**LTNP 009**	**LTNP 010**	**LTNP 011**
Nadir CD4	701	343	462	784	360	350	735	497	400	706	160
Years with HIV	15.91	22.79	31.07	18.81	14.23	24.03	10.37	15.71	17.14	13.69	25.8
CD4 at inclusion	1000	1300	1100	1180	410	480	951	506	460	1070	295*
VL at inclusion	19	19	19	19	1987	49	400	20	707	43	22
Age	70	58	71	52	37	72	52	36	68	57	67
Gender	F	M	M	M	F	F	F	F	M	F	M
Ethnicity	C	C	C	C	A	C	A	C	C	A	C

Each NCART number corresponds to the paired EC/LTNP number. * LTNP 011 did not fulfill all criteria due to decline in CD4 count after twelve years of infection, however still controlling plasma virus for fourteen more years. Caucasian (C), African (A), Female (F), Male (M), antiretroviral treatment (ART). Viral load (VL) measured as copies/mL, CD4 count measured as cells/µL. *CCR5* genotype: wild type (WT), 32-base-pair deletion (Δ32).

Supplementary Table 2: MHC II alleles in ECs and LTNPs.

Patient	Allele	HLA-DPA1	HLA-DPB1	HLA-DQA1	HLA-DQB1	HLA-DRB1
EC	1	DPA1*01:03:01	DPB1*02:01:02	DQA1*01:02:01	DQB1*06:03:01	DRB1*13:01:01
001	2	DPA1*01:03:01	DPB1*03:01:01	DQA1*01:03:01	DQB1*06:04:01	DRB1*13:02:01
EC	1	DPA1*01:03:01	DPB1*04:02:01	DQA1*01:03:01	DQB1*02:02:01	DRB1*07:01:01
002	2	DPA1*02:01:01	DPB1*17:01	DQA1*02:01:01	DQB1*06:03:01	DRB1*13:01:01
EC	1	DPA1*01:03:01	DPB1*04:01:01	DQA1*02:01:01	DQB1*03:02:01	DRB1*04:01:01
003	2	DPA1*01:03:01	DPB1*04:01:01	DQA1*03:01:01	DQB1*03:03:02	DRB1*07:01:01
EC	1	DPA1*01:03:01	DPB1*04:01:01	DQA1*01:02:01	DQB1*03:02:01	DRB1*04:01:01
004	2	DPA1*01:03:01	DPB1*04:01:01	DQA1*03:01:01	DQB1*06:02:01	DRB1*15:01:01
LTNP	1	DPA1*01:03:01	DPB1*01:01:01	DQA1*01:02:01	DQB1*03:01:01	DRB1*13:02:01
005	2	DPA1*02:01:08	DPB1*02:01:02	DQA1*05:05:01	DQB1*06:09:01	DRB1*13:03:01
LTNP	1	DPA1*01:03:01	DPB1*04:01:01	DQA1*02:01:01	DQB1*02:02:01	DRB1*04:01:01
006	2	DPA1*01:03:01	DPB1*06:01:01	DQA1*03:03:01	DQB1*03:01:01	DRB1*07:01:01
LTNP	1	DPA1*01:03:01	DPB1*02:01:02	DQA1*01:02:01	DQB1*06:02:01	DRB1*13:02:01
007	2	DPA1*03:01	DPB1*105:01:01	DQA1*01:02:01	DQB1*06:09:01	DRB1*15:03:01
LTNP	1	DPA1*01:03:01	DPB1*04:02:01	DQA1*01:02:01	DQB1*02:01:01	DRB1*03:01:01
008	2	DPA1*02:06	DPB1*05:01:01	DQA1*05:01:01	DQB1*06:02:01	DRB1*15:01:01
LTNP	1	DPA1*01:03:01	DPB1*04:01:01	DQA1*01:02:01	DQB1*03:01:01	DRB1*11:04:01
009	2	DPA1*01:03:01	DPB1*06:01:01	DQA1*05:05:01	DQB1*06:02:01	DRB1*15:01:01
LTNP	1	DPA1*02:01:01	DPB1*01:01:01	DQA1*02:01:01	DQB1*02:01:01	DRB1*03:01:01
010	2	DPA1*02:01:01	DPB1*17:01	DQA1*05:01:01	DQB1*02:02:01	DRB1*13:03:01
LTNP	1	DPA1*01:03:01	DPB1*04:01:01	DQA1*01:04:01	DQB1*03:02:01	DRB1*04:04:01
011	2	DPA1*01:03:01	DPB1*06:01:01	DQA1*03:01:01	DQB1*05:03:01	DRB1*14:54:01

Supplementary Table 3: Genes associated with HIV or innate sensing based on literature and IVA.

actr2 (ACTR2)	CSF2	HSPA9	ITGA3	OASL (OASL)	TAB1 (TAB1)	Trav5d-4
acyclovir	CTNNB1 (CTNNB1)	HTATSF1	ITGAL	P2X7 (P2RX7)	TAB2 (TAB2)	Trav6-5
AIF1 (AIF1)	CTSB (CTSB)	ICAM1 (ICAM1)	ITGB1	PANX1 (PANX1)	TAB3 (TAB3)	Trav6-7-dv9
AIM2 (AIM2)	CTSG (CTSG)	IFI16 (IFI16)	ITGB2	PCSK5	TACC2 (TACC2)	Trav6d-4
ALB (ALB)	CX3CR1 (CX3CR1)	IFIH1 (IFIH1)	KHNYN	pfn1 (PFN1)	TARBP1	Trav7-4
AP1M1	CXCL12 (CXCL12)	IFIT1 (IFIT1)	KLRC4-KLRK1/KLRK1	phorbol myristate acetate	TBK1 (TBK1)	Trav7-6
AP3D1	CXCR4 (CXCR4)	IFIT2 (IFIT2)	KLRK1 (Klrk1)	phosphatidylserine	TBL1XR1	Trav7d-5
AP3M1	CYBB (CYBB)	IFIT3 (IFIT3)	KPNA4	PIK3C2A	T-cell alpha/beta receptor	Trav7n-4
APOA5 (APOA5)	cyclosporin A	IFITM3 (IFITM3)	LapTM5	PIK3C2B	TCF4	Trav7n-6
APOBEC3F	DDOST (DDOST)	IFNa (IFNA17)	LCK	PIK3C2G	Tcrd	Trav8-1
APOBEC3G	DDX41 (DDX41)	IFNa (IFNA2)	LGALS1	PIK3C3	TFAP4	Trav8d-2
APP	DDX58 (DDX58)	IFNA1	lipopolysaccharide	PIK3CA	TFCP2	Trav8n-2
ASF1A	DDX60 (DDX60)	IFNA1 (IFNA4)	lmna (LMNA)	PIK3CB	TGFB1 (TGFB1)	Trav9-1
ATE1 ? (unmapped)	DEFA1 (+others)	IFNA10 (IFNA10)	lovastatin	PIK3CD	TGFB2 (TGFB2)	Trav9d-1
Atg12 (ATG12)	DEFB104A/DEFB104B	IFNA13	LRRFIP1	PIK3CG	TGFB3 (TGFB3)	TRB
Atg16L2 (ATG16L2)	Defb4/Defb5	IFNA14 (IFNA14)	LSP1	PIK3R1	thalidomide	Trbc1
Atg7 (ATG7)	DEFB4A/DEFB4B	IFNA16 (IFNA16)	luminespib	PIK3R2	TLR (unmapped)	Trbc2
B230359F08Rik	D-glucose	IFNA17 (IFNA17)	LYN	PIK3R3	TLR2 (TLR2)	Trbd1
BLK	disulfiram	IFNA2 (IFNA2)	LYST (LYST)	PIK3R4	TLR3 (TLR3)	Trbd2
bryostatin 1	DPM1 (DPM1)	IFNA21 (IFNA21)	MAP1A	PIK3R5	TLR4 (TLR4)	Trbj1-2
BST2	dstn (DSTN)	IFNA4 (IFNA4)	MAP1S	PIK3R6	TLR7 (TLR7)	Trbj2-5
butyric acid	dstn (Dstn/Dstnl1)	IFNA5 (IFNA5)	MAP3K7 (MAP3K7)	PPIA	TLR8 (TLR8)	Trbv1
C4BPA (C4BPA)	duvoglustat	IFNA6 (IFNA6)	MAPK1	PPIA (PPIA)	TLR9 (TLR9)	Trbv12-1
CASP1 (CASP1)	EBF2 (EBF2)	IFNA7 (IFNA7)	MAPK11	PRKACA	TMEM173	Trbv13-1
casp10 (CASP10)	EGF	IFNA8 (IFNA8)	MAPK12	PRKACB	tmod1 (TMOD1)	Trbv13-2
casp2 (CASP2)	ELANE (ELANE)	IFNAR1	MAPK13	PRKACG	TNC	Trbv13-3
CASP3 (CASP3)	EZH2	IFNb (IFNB1)	MAPK14	PRKCA	TNF	Trbv14
CASP4 (CASP4)	FAS (FAS)	IFNB1 (IFNB1)	MAPK3	PRKDC (PRKDC)	TNF (TNF)	Trbv15
CASP5 (CASP5)	FCER1G	IFNG (IFNG)	MAPKs (unmapped)	PRNP	TNFRSF1A	Trbv16
CASP6 (CASP6)	FCGR1A	IFNK (IFNK)	MAVS (MAVS)	PSIP1 (PSIP1)	TNPO3	Trbv17
casp7 (CASP7)	FCGR1B	IFNL1 (IFNL1)	MB21D1 (MB21D1)	PTPN11	TNPO3 (TNPO3)	Trbv19
casp8 (CASP8)	FCGR2A	IFNL2 (IFNL2)	MBL2 (MBL2)	PTPN6	TRA	TRBV19
casp9 (CASP9)	FCGR2B	IFNL3 (IFNL3)	Med14 (LOC103694537)	PYCARD (PYCARD)	Trac	Trbv2
cat (CAT)	FCGR2C	IFNW1 (IFNW1)	Med14 (MED14)	Rab6A (RAB6A)	TRAF3 (TRAF3)	Trbv20
cat (CRAT)	FCGR3A/FCGR3B	Ifnz (+others)	Med28 (MED28)	RABEPK (RABEPK)	TRAF6 (TRAF6)	Trbv23
cat (GLYAT)	fgd6 (FGD6)	IGF1R (IGF1R)	Med4 (MED4)	RanBP2 (RANBP2)	TRAPPC1	Trbv26
cat (GOT1)	FGR	IGF2BP1	Med6 (MED6)	RanBP2 (RGPD4)	Trav1	Trbv3
cat (GOT2)	FN1	IgG1	Med7 (MED7)	RBBP7	Trav10	Trbv30
cat (MIP)	FOLH1 (FOLH1)	IgG1 kappa	MICA (MICA)	REL	Trav10d	Trbv31
cat (TRPV6)	FOXP3	IgG2a	miRNA-155 (miR-155-5p)	RELA (RELA)	Trav10n	Trbv4
CCAR2 (CCAR2)	FRK	Igg3	miRNA-2337 (unmapped)	RELB	Trav11	Trbv5
CCL11	FUT2 (FUT2)	IgG4	MKKS (MKKS)	Rgp1p ? (unmapped)	Trav11d	TRD
CCL13	FYN	IGH	MMP9	RNASEL (RNASEL)	Trav12-2	TREX1
CCL2	GABARAPL2	IGHG1	morphine	RNF19A (RNF19A)	Trav12-3	TRG
CCL24	GPR1	IGHG2	MOV10	ryr2 (RYR2)	Trav12d-3	TRGV9
CCL26	GRM3 (GRM3)	Ighg2b	MST1R	S100A8 (S100A8)	Trav12n-2	trichostatin A
CCL3	gzmb (GZMB)	IGHG3	MUC5B	S100A9 (S100A9)	Trav13-2	TRIM22
CCL4	GZMH (GZMH)	IGHG4	MX1 (MX1)	SAMHD1 (SAMHD1)	Trav13-4-dv7	TRIM32
CCL5 (CCL5)	HCK	IGK	MX2 (MX2)	SAP130	Trav13d-4	TRIM49 /TRIM49C
CCNT1 (CCNT1)	HDAC1	IGL	MYD88 (MYD88)	SAP18	Trav13n-4	TRIM5
CCR2 (CCR2)	HDAC10	IKBKB (IKBKB)	NEDD4L	SAP30	Trav14-3	TRIM64/TRIM64B
CCR5 (CCR5)	HDAC11	IKBKG (IKBKG)	NFkB (family)	SAP30L	Trav14d-1	TSG101
CD209	HDAC2	IL10 (IL10)	NFKB1 (NFKB1)	SDR39U1 (SDR39U1)	Trav14d-3-dv8	UBB
CD247	HDAC3	IL13	NfkB1-RelA	SERPINA1	Trav14n-2	Ubb
CD28	HDAC4	IL19 (IL19)	NFKB2	SFTPD	Trav15-1-dv6-1	UBC
CD3	HDAC5	IL1B (IL1B)	NFKBIA (NFKBIA)	SIGIRR (SIGIRR)	Trav15-2-dv6-2	UBD
CD3D	HDAC6	IL1R1 (IL1R1)	NfkB-RelA	SIN3A	Trav15d-1-dv6d-1	valacyclovir
CD3E	HDAC7	IL1RAP (IL1RAP)	NLRP1 (NLRP1)	SIN3B	Trav15d-2-dv6d-2	vorinostat
CD3G	HDAC8	IL2	NLRP3 (NLRP3)	SLFN11 (FLJ34922	Trav15n-1	Vps proteins class E
CD4 (CD4)	HDAC9	IL20 (IL20)	NLRP6 (NLRP6)	slx4 (SLX4)	Trav16	VPS28
Cdk9 (CDK9)	HIRA	IL23R (IL23R)	NMT1 (NMT1)	SMCIA (unmapped)	Trav16d-dv11	VPS4A
CHD1	HLA-A (unmapped)	IL3	NOD2 (NOD2)	SNORD37 (SNORD37)	Trav17	VPS4B
CHMP2A	HLA-B (unmapped)	IL37 (IL37)	NOVA1 (NOVA1)	SNORD37 (SNORD37)	Trav18	Vps53
CHORDC1	HLA-C (unmapped)	IL6 (IL6)	NSMCE4A (NSMCE4A)	SNORD37 (SNORD37)	Trav19	WRN
CHUK (CHUK)	hnRNPA1 (HNRNPA1)	IL7	nup107 (NUP107)	SRC	Trav2	XRCC5
ckap4 (CKAP4)	HNRNPCL1/HNRNPCL2	imiquimod	Nup133 (NUP133)	SRSF1	Trav21-dv12	XRCC6
CLEC4A	HNRNPF (HNRNPF)	interferon beta-1a	Nup153 (NUP153)	STAT1 (STAT1)	Trav3-3	YES1
CLEC4M	HSPA14	IRAK1 (IRAK1)	Nup160 (NUP160)	stmn1 (STMN1)	Trav3-4	YWHAE
CLN3 (CLN3)	HSPA1A/HSPA1B	IRAK2 (IRAK2)	NUP62	STT3A (STT3A)	Trav3d-3	YWHAH
CLU	HSPA1L	IRAK4 (IRAK4)	Nup85 (NUP85)	STX5 (STX5)	Trav3n-3	YWHAQ
CMA1 (CMA1)	HSPA2	IRF1 (IRF1)	NYNRIN (NYNRIN)	STXBP6 (STXBP6)	Trav4-3	ZAP70
CNP	HSPA4	IRF3 (IRF3)	NAALDA2 (unmapped)	SUMO1	Trav4-4-dv10	ZBP1 (ZBP1)
COG (TG)	HSPA5	IRF7 (IRF7)	OAS1 (OAS1)	SUPT16H	Trav4d-3	ZNRD1
CR2 (CR2)	HSPA6	IRF8	OAS2 (OAS2)	SUPT6H	Trav4n-4	
CREBZF	HSPA8	ITGA2	OAS3 (OAS3)	SYK	Trav5-4	

523 genes represented in the biological filter in the IVA filtering process. The genes are associated with HIV or innate immune sensing and signaling based on the literature, or associated with HIV according to IVA.

Supplementary table 4: Additional information on genetic variants identified in the EC and LTNP cohort

Patient number	Gene Symbol	Gene name	RVIS	Z	pLI	dbSNP ID	Position	Transcript ID
EC 001								
EC 002								
EC 003								
EC 004	*DDOST*	*dolichyl-diphosphooligosaccharide--protein glycosyltransferase non-catalytic subunit*	21%	0.36	0.13	145940009	chr1:20979136	NM_005216.4
	TAB2	*TGF-beta activated kinase 1/MAP3K7 binding protein 2*	7%	1.06	1		chr6:149718863	NM_015093.5
	MMP9	*matrix metallopeptidase 9*	62%	1.96	0	199533749	chr20:44643062	NM_004994.2
LTNP 005	*PIK3C2B*	*phosphatidylinositol-4-phosphate 3-kinase catalytic subunit type 2 beta*	13%	1.72	0.98	143689889	chr1:204408074	NM_002646.3
	FRK	*fyn related Src family tyrosine kinase*	67%	0.15	0	146191498	chr6:116265462	NM_002031.2
LTNP 006	*EGF*	*epidermal growth factor*	40%	0.22	0	746246476	chr4:110909839	NM_001963.5
	MAP1A	*microtubule associated protein 1A*	59%	0.42	1	201773895	chr15:43821880	NM_002373.5
	PIK3R5	*phosphoinositide-3-kinase regulatory subunit 5*	60%	2.24	0.82		chr17:8808991	NM_001142633.2
LTNP 007	*FGD6*	*FYVE, RhoGEF and PH domain containing 6*	73%	-0.59	1	139181168	chr12:95604924	NM_018351.3
	CMA1	*chymase 1*	92%	-3.03	0	138432864	chr14:24976674	NM_001836.4
LTNP 008	*FN1*	*fibronectin 1*	13%	1.39	0.06	147831535	chr2:216284009	NM_212482.2
	LRRFIP1	*LRR binding FLII interacting protein 1*	68%	-1.08	0.37	200750365	chr2:238661991	NM_004735.3
	IRAK2	*interleukin 1 receptor associated kinase 2*	97%	-0.26	0	368608528	chr3:10268107	NM_001570.3
	PRKDC	*protein kinase, DNA-activated, catalytic polypeptide*	-	0.03	1	202110076	chr8:48792167	NM_006904.6
	MED6	*mediator complex subunit 6*	28%	0.5	0.01		chr14:71058039	NM_001284209.1
	NOD2	*nucleotide binding oligomerization domain containing 2*	98%	-0.88	0	61755182	chr16:50741791	NM_022162.2
LTNP 009	*FN1*	*fibronectin 1*	13%	1.39	0.06	148505961	chr2:216226780	NM_212482.2
	FN1	*fibronectin 1*	13%	1.39	0.06	139452116	chr2:216240047	NM_212482.2
	PIK3R6	*phosphoinositide-3-kinase regulatory subunit 6*	-	-1.09	0	201062021	chr17:8706748	NM_001010855.3
LTNP 010	*DDOST*	*dolichyl-diphosphooligosaccharide--protein glycosyltransferase non-catalytic subunit*	21%	0.36	0.13	150551993	chr1:20980798	NM_005216.4
	SLX4	*SLX4 structure-specific endonuclease subunit*	99%	-2.23	0	758409623	chr16:3639204	NM_032444.3
	PRKDC	*protein kinase, DNA-activated, catalytic polypeptide*	-	0.03	1	55644332	Chr8:48733399	NM_006904.6
LTNP 011	*CCNT1*	*cyclin T1*	49%	1.1	1	61751602	chr12:49087282	NM_001240.3
	PRKCA	*protein kinase C alpha*	5%	3.5	0.95	373125854	chr17:64728917	NM_002737.2

Additional information on variants identified in the EC and LTNP cohort (shown in Table 3.) Residual variance intolerance score (RVIS): percentages of all genes that are more likely to be intolerant. The Exome Aggregation Consortium (ExAC) database constraint values for missense variants: Positive Z score indicates that the gene is intolerant to variation, and a negative value shows that the gene contains more variants than expected; a pLI score for loss of function variants ≤0.1 indicates complete tolerance to variants, and pLI≥0.9 indicates an extremely intolerant gene. Single nucleotide polymorphism database identification number (dbSNP ID).

Supplementary table 5: Variants identified in a Herpes Simplex Encephalitis (HSE) cohort including 11 patients

Patient	Position	Region	Gene Symbol	Transcript	Transcript Variant	Protein Variant	CADD	MSC	PP2	dbSNP ID	Gnom AD
HSE1	chr11:116661001	Exonic	*APOA5*	NM_052968.4	c.944C>T	p.A315V	28.0	<1	Po.D	143292359	0.059
	chr22:42033662	Exonic	*XRCC6*	NM_001469.4	c.640T>C	p.S214P	29.0	3.3			
HSE2											
HSE3											
HSE4	chr3:10254969	Exonic	*IRAK2*	NM_001570.3	c.607G>A	p.V203M	26.6	3.3	B	148608407	0.100
HSE5											
HSE6											
HSE7	chr1:161496010	Exonic	*HSPA6*	NM_002155.4	c.1562T>G	p.V521G	22.40	3.3	Pr.D	199677197	0.218
	chr11:5686140	Exonic	*TRIM5*	NM_033034.2	c.1381A>C	p.I461L	26.2	3.3	Po.D	142925424	0.084
	chr18:61071041	Exonic	*VPS4B*	NM_004869.3	c.383G>A	p.R128Q	28.0	5.3	B	373315578	0.000
HSE8	chr12:18552594	Exonic	*PIK3C2G*	NM_004570.5	c.2005C>T	p.L669F	31.0	3.3	Pr.D	61754413	0.337
	chr17:8725232	Exonic	*PIK3R6*	NM_001010855.3	c.1810C>T	p.R604*	37.0	3.3		373852885	0.009
HSE9	chr1:182555149	Exonic	*RNASEL*	NM_021133.3	c.793G>T	p.E265*	35.0	21.1		74315364	0.359
	chr1:182555767	Exonic	*RNASEL*	NM_021133.3	c.175G>A	p.G59S	25.3	21.1	Pr.D	151296858	0.353
	chr17:47118862	Exonic	*IGF2BP1*	NM_006546.3	c.941C>T	p.S314L	24.6	5.7	B	368120375	0.003
	chr16:50746086	Exonic	*NOD2*	NM_022162.2	c.2264C>T	p.A755V	24.4	<1	Pr.D	61747625	0.251
	chr4:187003729	Exonic	*TLR3*	NM_003265.2	c.889C>G	p.L297V	23.3	19.6	Pr.D	35311343	0.155
HSE10	chr12:49087306	Exonic	*CCNT1*	NM_001240.3	c.1691C>T	p.S564F	25.7	3.3	B	752700019	0.054
	chrX:48682140	Exonic	*HDAC6*	NM_006044.3	c.3248G>A	p.G1083D	26.4	3.3	Pr.D	41312114	0.172
	chr1:182555149	Exonic	*RNASEL*	NM_021133.3	c.793G>T	p.E265*	35.0	21.1		74315364	0.359
	chr1:182555767	Exonic	*RNASEL*	NM_021133.3	c.175G>A	p.G59S	25.3	21.1	Pr.D	151296858	0.353
HSE11	chr12:18552594	Exonic	*PIK3C2G*	NM_004570.5	c.2005C>T	p.L669F	31.0	3.313	Pr.D	61754413	0.337

No variants were identified in HSE patient number 2, 3, 5, and 6. Identical variants are marked with green. Genes that are also affected in the EC and LTNP cohort are marked with yellow. Mutation Significance Cutoff (MSC) score for the Combined Annotation Dependent Depletion (CADD) score; splice cite loss (SSL). PolyPhen-2 function prediction (PP2); probably damaging (Pr.D); possibly damaging (Po.D); and benign (B). The Genome Aggregation Database (GnomAD) frequency. Full gene designations: *apolipoprotein A5 (APOA5), X-ray repair cross complementing 6 (XRCC6), interleukin 1 receptor associated kinase 2 (IRAK2), heat shock protein family A member 6 (HSPA6), Tripartite Motif Containing 5 (TRIM5), Vacuolar Protein Sorting 4 Homolog B (VPS4B), Phosphatidylinositol-4-Phosphate 3-Kinase Catalytic Subunit Type 2 Gamma (PIK3C2G), phosphoinositide-3-kinase regulatory subunit 6 (PIK3R6), ribonuclease L (RNASEL), Insulin Like Growth Factor 2 MRNA Binding Protein 1 (IGF2BP1), nucleotide binding oligomerization domain containing 2 (NOD2), TLR3, cyclin T1 (CCNT1), and Histone Deacetylase 6 (HDAC6).*

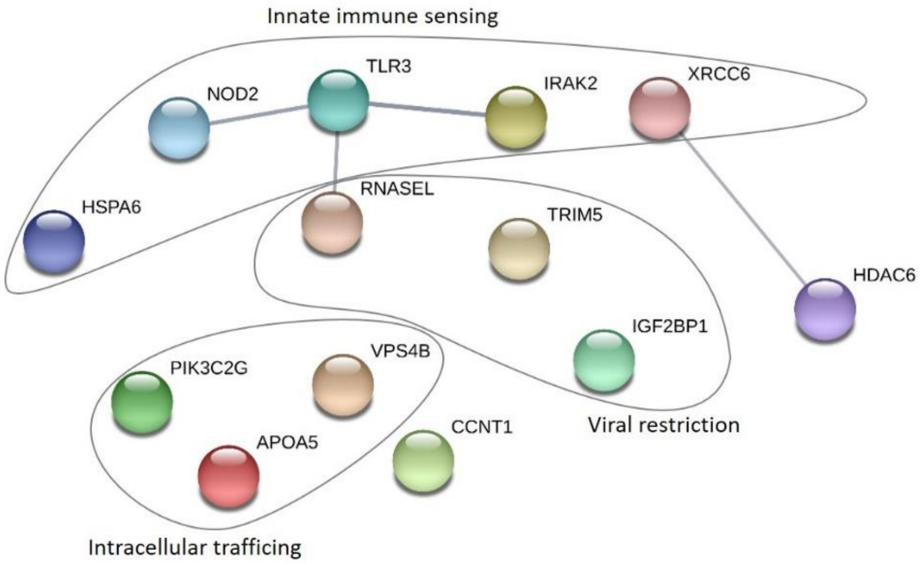

Supplementary Figure 1: STRING association network for variants identified in the HSE cohort. Protein-protein interaction network for proteins encoded by genes with variants within the HSE cohort. *PIK3R6* is not a part of the network due to absence from the database. Each circle represents a gene/protein affected by at least one variant in the cohort. Thickness of gray lines represents strength of data supporting association, i.e. proteins jointly contribute to a shared function. Interactions are made with STRING version 10.5 based on data from genomic context predictions, high-throughput experiments, co-expressions, automated text-mining and database search. Circles are drawn around proteins contributing to similar pathways or biological functions. The PPI enrichment p-value for the number of identified edges (4) compared to expected (2) in a group of 13 proteins was 0.0725, thus not significantly different from expected, with a minimum interaction score a 0.4.

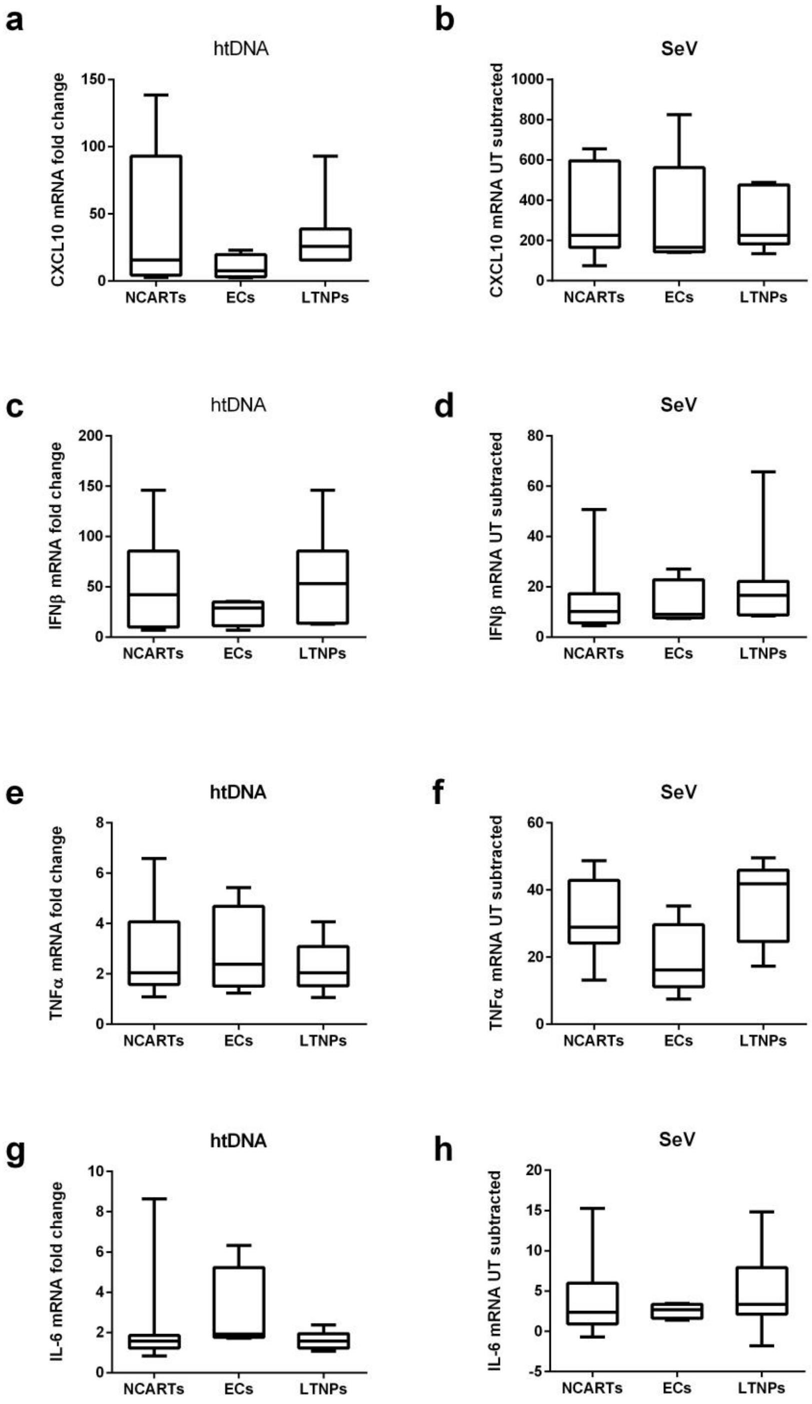

Supplementary figure 2: Innate immune response in ECs, LTNPs, and NCARTs at cohort level.

PBMCs were transfected with herring testes (ht)DNA (2 μg/mL) using Lipofectamine 3000 (A,C,E,G) or infected with sendai virus (SeV) (1:500) (B,D,F,H) for 6 hrs, after which cells were lysed and RNA was isolated and used for qPCR. The mRNA expressions of IFNβ (AB), CXCL10 (C,D), TNFα (E,F), and IL-6 (G,H)

were normalized to TBP. Median with 95 percentile and error bars representing min-max values are shown. Non-controllers on ART (NCARTs); elite controllers (ECs); long-term non-progressors (LTNPs); untreated (UT).
